# *Ligilactobacillus salivarius* CECT5713 Increases Term Pregnancies in Women with Infertility of Unknown Origin: A Randomized, Triple-Blind, Placebo-Controlled Trial

**DOI:** 10.3390/nu17111860

**Published:** 2025-05-29

**Authors:** Cristina Huerga López, María J. Sánchez Martín, Aránzazu Herráez Moreta, Marta Calvo Urrutia, Ignacio Cristóbal García, Cristina Díaz Morillo, Ruth Blanco-Rojo, María E. Sáez, Mónica Olivares, Rebeca Arroyo, Carmen Herranz, Claudio Alba, Juan M. Rodríguez, Leonides Fernández

**Affiliations:** 1 Hospital Clínico San Carlos, 28040 Madrid, Spain; cristinahuergalopez@gmail.com (C.H.L.); chus1901@gmail.com (M.J.S.M.); aherraezmoreta@gmail.com (A.H.M.); marta.urrutia@salud.madrid.org (M.C.U.); ignacio.cristobal@salud.madrid.org (I.C.G.); 2 Kerry Group, Camino de Purchil 66, 18004 Granada, Spain; cristina.diaz@kerry.com (C.D.M.); ruth.blanco@kerry.com (R.B.-R.); mariu.saez@kerry.com (M.E.S.); monica.maria.olivares@kerry.com (M.O.); 3 Department of Nutrition and Food Science, Complutense University of Madrid, 28040 Madrid, Spain; rebecaa@ucm.es (R.A.); cherranz@ucm.es (C.H.); c.alba@ucm.es (C.A.); jmrodrig@ucm.es (J.M.R.); 4 Instituto Pluridisciplinar, Complutense University of Madrid, 28040 Madrid, Spain; 5 Department of Galenic Pharmacy and Food Technology, Complutense University of Madrid, 28040 Madrid, Spain

**Keywords:** *Ligilactobacillus salivarius*, infertility, probiotics, assisted reproduction techniques, vaginal microbiota, semen microbiota, TGFβ1, VEGF

## Abstract

**Background/Objectives:** Unexplained infertility is a worldwide problem affecting a significant proportion of couples of reproductive age. Recent studies suggest that alterations in the vaginal microbiota are related to female infertility, while supplementation with some probiotic strains has been shown to improve pregnancy rates in couples experiencing this problem. This study aimed to evaluate the impact of oral administration of *Ligilactobacillus salivarius* CECT5713 on pregnancy success rates in couples with unexplained infertility prior to in vitro fertilization (IVF). **Methods**: Seventy couples were randomized to receive either a placebo or a probiotic intervention (one capsule per day containing an excipient only or 3 × 10^9^ viable cells of *L. salivarius* CECT5713 plus an excipient, respectively); 57 couples completed the study. Baseline data on demographics, health status (including gynecological and reproductive history), and lifestyle habits were collected. Vaginal swabs and semen samples were obtained from each couple before the intervention and immediately prior to IVF or upon confirmed pregnancy and were analyzed for microbiological (using both culture-dependent and -independent methods) and immunological profiles. **Results**: Oral administration of *L. salivarius* CECT5713 in couples with unexplained infertility scheduled for IVF resulted in a significantly higher pregnancy success rate (48.1%) compared to the placebo group (20.0%) (one-tailed Chi-square test; *p* < 0.024). The probiotic intervention improved both vaginal and semen immunological profiles, with no substantial changes observed in their microbial composition. **Conclusions**: These preliminary findings support the potential of *L. salivarius* CECT5713 supplementation to enhance fertility outcomes in couples with unexplained infertility.

## 1. Introduction

Infertility is a growing issue worldwide [1], affecting approximately 10–14% of couples of reproductive age [2,3]. This condition is defined as the failure to achieve a clinical pregnancy after 12 months or more of regular unprotected sexual intercourse [4]. While there are many recognized causes of infertility, such as ovulatory dysfunction, tubal factors, male-related factors, and problems with the ovaries or uterus, around 15–25% of couples seeking fertility treatment still have unexplained reasons for their infertility, referred to as “unexplained infertility” [3].

Some studies have shown a negative correlation between a *Lactobacillus*-dominated vaginal microbiota, characterized by low diversity and high *Lactobacillus* concentration, and female infertility [5,6,7], highlighting the relevance of the microbiota of the female genital tract for human reproduction [8,9,10]. In this frame, probiotics have been postulated as additional tools to improve fertility outcomes [11,12,13], given the limited efficacy of available treatments for recurrent abortion and unexplained infertility [14,15]. In practice, the empirical use of commercial probiotics as a complementary treatment for women with unexplained infertility is increasing [9,10], despite the limited scientific or clinical evidence supporting their global usefulness for this target [16]. The efficacy of probiotics for any specific application depends on the strains, the posology, and the delivery method. Therefore, a careful case-by-case evaluation of probiotics with the potential to contribute to the fertility field is necessary [16].

In a previous trial, oral administration of *Ligilactobacillus salivarius* CECT5713 to women experiencing either repetitive abortion or infertility of unknown origin led to a significant increase in pregnancy rates in both groups [17]. This strain was selected for this application due to its vaginal-related properties, including its remarkable acidifying activity through the production of high amounts of L-lactate, its α-amylase activity, its strong adhesiveness to vaginal cells, and its antimicrobial activity against vaginal pathogens. Additionally, it demonstrated a high survival rate when exposed to conditions resembling those of the human gastrointestinal tract and was proven safe when administered to lactating women and children [17,18,19,20]. However, this proof-of-concept trial was designed as an open-label, non-placebo-controlled study and, in addition, included women who were not programmed for assisted reproduction therapies.

In this context, the main objective of this study was to evaluate the impact of oral administration of *L. salivarius* CECT5713 on the pregnancy success rate of couples with unexplained infertility prior to undergoing in vitro fertilization (IVF) procedures.

## 2. Materials and Methods

### 2.1. Study Design

This study was a non-inferiority randomized triple-blind placebo-controlled trial that compared the effectiveness of oral probiotic supplementation with *L. salivarius* CECT5713 versus placebo in a regular IVF procedure in couples with unexplained infertility to obtain a successful pregnancy (live birth).

The study was approved by the Ethics Committee of the Hospital Clínico San Carlos (Madrid, Spain) (reference 20/168-EC_X, date of approval: 18 March 2020, act 3.2/20) and conducted according to the ethical principles addressed in the Declaration of Helsinki and Good Clinical Practice. The study was registered in the ClinicalTrials.gov database (NCT06290518).

### 2.2. Sample Size Estimation

The sample size was estimated based on the IVF success rate at the Assisted Reproduction Service at the Hospital Clínico San Carlos (Madrid, Spain) for unexplained infertility (32%) and preliminary data from a pilot study with *L. salivarius* CECT5713, in which 56% of women with reproductive failure achieved successful pregnancies [17]. A non-inferiority margin of 10%, with 80% power and a 2.5% level of significance (one-sided test), resulted in an estimated sample size of 32 in each group. The sample size estimation was performed using a web application for testing non-inferiority for two parallel-sample proportions (https://www2.ccrb.cuhk.edu.hk/stat/proportion/tspp_sup.htm, accessed on 20 January 2020) and according to the method of Farringtong and Manning [21]. Considering a maximum admissible drop-out rate of 20%, the number of couples to be enrolled, according to Freedman’s formula, was 70.

### 2.3. Study Population

Volunteers were recruited among couples attending the Assisted Reproduction Service at the Hospital Clínico San Carlos (Madrid, Spain) with unexplained infertility and indication for IVF. The inclusion criteria were as follows: adults of legal age intending to achieve pregnancy for at least 12 months but unable to do so due to an unknown cause, willingness to undergo IVF treatment, and being on the waiting list for an IVF cycle with an expected waiting time of more than 5 months. Participants were excluded at the time of enrollment if one member of the couple was sterile (i.e., azoospermia, fallopian tube blockage), had genitourinary malformations, any other severe diseases (i.e., cancer, Acquired Immune Deficiency Syndrome (AIDS), Amyotrophic Lateral Sclerosis (ALS), morbid obesity) or uncontrolled chronic diseases (i.e., inflammatory bowel disease, diabetes), planned treatment or intervention other than IVF within the 12 weeks following the start date of the intervention, were on chronic antibiotic treatment, were consuming or intended to consume another probiotic supplement within the following 3 months, participate in another clinical trial, had an allergy or intolerance to the probiotic excipient, or showed an inability to understand the informed consent form and/or follow basic trial instructions. Couples who withdrew from the study were not replaced.

Participants were enrolled from 17 November 2020 to 22 November 2022. The last embryo transfer was performed on 6 June 2023. Written informed consent was obtained from each participant couple.

### 2.4. Randomization

Simple random allocation to either of the two study arms (placebo or probiotic group) was carried out by a statistician who had no other involvement with the study, using a computer-generated random number generator. Allocation of the participants to receive the probiotic or only the excipient was conducted using sequentially numbered, sealed, and opaque envelopes. The patients, doctors, and laboratory team were blind regarding the assigned group.

### 2.5. Study Intervention

The volunteer couples who met the inclusion criteria and were assigned to the probiotic group received, both man and woman, one daily capsule containing a total of 3 × 10^9^ viable cells of *L. salivarius* CECT5713 plus maltodextrin as excipient, while those assigned to the placebo group received one daily capsule containing only the excipient (maltodextrin). Capsules containing the probiotic and the excipient or only the excipient were indistinguishable. The probiotic or the excipient was administered after the first visit, during the six previous months prior to the standard IVF procedure, and also during the first IVF cycle (1–2 months). In the event of pregnancy, either before or after the IVF, women continued taking the product (either the probiotic or the excipient) during the first 12 weeks of pregnancy. Adherence to the intervention was recorded in daily diaries. If the participants did not take the probiotic or placebo for two or more days per week (intake of <85% of the recommended dose), it was considered a protocol deviation.

Baseline data for demographics, participant characteristics, health status, including gynecological history and reproductive data, past and actual infectious and autoimmune diseases, drug treatments, and healthy habits, were gathered during the first visit after recruitment. For each couple, a vaginal swab and a semen sample were collected for both groups before starting the intervention (time 1) and, also, just before starting the IVF cycle, or at any time before if the woman attained pregnancy (whichever happened first, time 2). The vaginal swabs and semen samples were kept at −20 °C at the hospital and transported to the laboratory of the Complutense University of Madrid under temperature-controlled conditions and then stored at −80 °C until microbiological and immunological analysis.

Periodic visits (phone or presential) were scheduled to record comedication, tolerance to the product (probiotic and/or excipient), and pregnancy progress and outcomes.

### 2.6. Primary and Secondary Outcomes of the Study

The primary outcome measure was the number of participants who achieved a successful pregnancy that resulted in a live baby without birth defects. The secondary outcomes included assessing all spontaneous and IVF-related pregnancies, reporting the IVF parameters, abortion rate, pregnancy and birth outcomes, and characterizing the microbial and immunological profiles of the vaginal and semen samples. This information would provide a more comprehensive understanding of the intervention’s impact. It would also allow for identifying potential microbiological and immunological markers that could help identify idiopathic infertile couples for whom the administration of *L. salivarius* CECT5713 might be effective in achieving a successful pregnancy.

### 2.7. Culture-Dependent Analysis of Vaginal Swabs and Semen Samples

All vaginal swabs and semen samples obtained at times 1 and 2 were submitted to culture-dependent analysis in Columbia Nalidixic Acid (CNA; BioMèrieux, Marcy l’Etoile, France), MacConkey (MCK), and de Man, Rogosa and Sharpe supplemented with L-cysteine (2.5 g/L) (MRS-C) agar plates. The inoculated plates were incubated at 37 °C for 24–48 h in an aerobic atmosphere, except those of MRS-C, which were incubated in an anaerobic atmosphere (85% N_2_, 10% H_2_, 5% CO_2_) in an anaerobic workstation MACS-MG-1000 (Don Whitley Scientific Ltd., Bingley, UK). Total viable and differential microbial counting were determined for each sample. A representative of each colony morphology was isolated, and after repeated streaking in agar plates to confirm its purity, a glycerol stock of each isolate was stored at −20 °C until identification. The isolates were grown in Brain Heart Infusion (BHI) or MRS broth and identified by matrix-assisted laser desorption–ionization time-of-flight mass spectrometry (MALDI-TOF MS; Vitek MS, BioMèrieux). Partial 16S rRNA gene sequencing was used for those isolates in which no identification was possible with the MALDI-TOF technique.

### 2.8. DNA Extraction from Vaginal Swabs and Semen Samples

DNA was extracted from vaginal swab suspensions in saline buffer (1 mL) and semen (1 mL) samples following the method described by Lackey et al. [22]. The samples were centrifuged at 11,000× *g* for 10 min at 4 °C. The QIAamp DNA Stool Mini Kit (Qiagen, Germantown, MD, USA) was used for DNA extraction, with additional steps for mechanical lysis and the removal of RNA and proteins using ribonuclease A and proteinase K, respectively. The sample DNA purity and concentration in the extracted DNA were measured with a NanoDrop 1000 spectrophotometer (NanoDrop Technologies, Inc., Rockland, DE, USA). Samples were stored at −20 °C until further analysis procedures.

### 2.9. Quantification of L. salivarius DNA in Vaginal Samples

*L. salivarius* DNA in vaginal swab suspensions was estimated using a real-time quantitative PCR assay and the method described by Harrow et al. [23] and adapted by Fernández et al. [17], which is based on the amplification of a 97-bp product of the 16S–23S intergenic spacer region of *L. salivarius*.

### 2.10. Metataxonomic Profiling

Metataxonomic profiling was carried out for both the vaginal and semen samples through high-throughput sequencing of the 16S rRNA gene using the Illumina MiSeq platform at the Madrid Science Park. Universal primers S-D-Bact-0341-b-S-17 (CCTACGGGNGGCWGCAG) and S-D-Bact-0785-a-A-21 (GACTACHVGGGTATCTAATCC), targeting the V3–V4 hypervariable regions of the 16S rRNA gene, were utilized [24].

In the secondary PCR, forward and reverse sequences were demultiplexed using unique barcodes appended to the 3′ and 5′ termini of the PCR amplicons. The DNA concentration for each sample was determined using the 2100 Bioanalyzer system (Agilent, Santa Clara, CA, USA). PCR products were pooled in equimolar concentrations, and the correct-sized fragments were excised from agarose gels, purified using the QIAEX II Gel Extraction Kit (Qiagen), and quantified using the PicoGreen assay (BMG Labtech, Jena, Germany). The pooled, purified, and barcoded amplicons were sequenced using the paired-end protocol on an Illumina MiSeq platform (Illumina Inc., San Diego, CA, USA) following the manufacturer’s protocols, with sequencing performed at the Madrid Science Park (Tres Cantos, Spain). Negative controls were included in all PCR reactions to exclude contamination.

Post-sequencing quality control was performed using the DADA2 pipeline. Paired-end reads were merged, and sequences were processed using QIIME software (version 2022.2) [25,26]. Taxonomic classification of operational taxonomic units (OTUs) was performed using the SILVA SSU database (version 138.1).

Alpha diversity, reflecting the richness and evenness of the vaginal and semen microbiota, was assessed using the Shannon and Simpson diversity indices. Beta diversity, representing differences in microbial composition between groups, was analyzed using permutational multivariate analysis of variance (PERMANOVA) based on Bray–Curtis and Jaccard distance matrices. These analyses evaluated the effects of intervention group (probiotic, placebo) and outcome (pregnancy, unsuccessful pregnancy) on the variation in microbial community structure.

### 2.11. Immunological Analyses in Vaginal and Semen Samples

The measurement of transforming growth factor β1 (TGFβ1) in the vaginal and semen samples and vascular endothelial growth factor (VEGF) in the vaginal samples was determined using RayBio^®^ Human TGF-β1 ELISA (RayBiotech, Norcross, GA, USA) and Human Cytokine VEGF Asssays (Bio-Rad, Hercules, CA, USA), respectively, following the manufacturer instructions. The samples were subjected to an acid treatment, followed by a neutralization step, before measuring the immunoreactive TGFβ1. The quantification of interleukin (IL)-2, IL-4, IL-6, IL-8, IL-10, granulocyte macrophage colony-stimulating factor (GM-CSF), interferon (IFN) γ, and tumor necrosis factor (TNF) α in the semen samples was performed using the Pro Human Cytokine 8-plex Assay (Bio-Rad) in the Bio-Plex 200 system (Bio-Rad).

### 2.12. Statistical Analysis

For the primary outcome, a one-tailed Chi-square test was performed at a significance level of 0.025 to compare the pregnancy rates that resulted in a live baby without birth defects between the placebo and probiotic groups. Absolute risk reduction (ARR) and 95% CI were calculated to assess the relationship between the administration of *L. salivarius* CECT5713 and the improvement in reproductive success (the primary outcome of the study).

Data were assessed for normality using the Shapiro–Wilk test. Parametric data were reported as mean values and 95% confidence intervals (CI), while non-parametric data were reported as median values and quartiles 1 and 3 (Q1, Q3). Parametric data were analyzed using a one-way analysis of variance (ANOVA) to compare the probiotic and placebo groups, or a paired *t*-test to compare the values before and after the intervention within the same group. When the data did not follow a normal distribution, the Wilcoxon rank-sum test was used to compare the probiotic and placebo groups, while the Wilcoxon signed-rank test was applied for within-group comparisons before and after the intervention. Fisher’s exact tests (or the Freeman–Halton extension for contingency tables larger than 2 × 2) and Chi-square tests were used to compare the categorical variables. A two-sided *p*-value < 0.05 was considered statistically significant without adjustment for multiplicity in the analysis of secondary outcomes or subgroup analyses, as they were considered and should be interpreted as exploratory.

The statistical analysis was performed using StatGraphics Centurion 19 (Statgraphics Technologies, Inc., The Plains, VA, USA) or the R environment (version 4.1.0; R-project, http://www.r-project.org, accessed on 22 May 2024).

## 3. Results

### 3.1. Study Randomization

**Figure 1:**
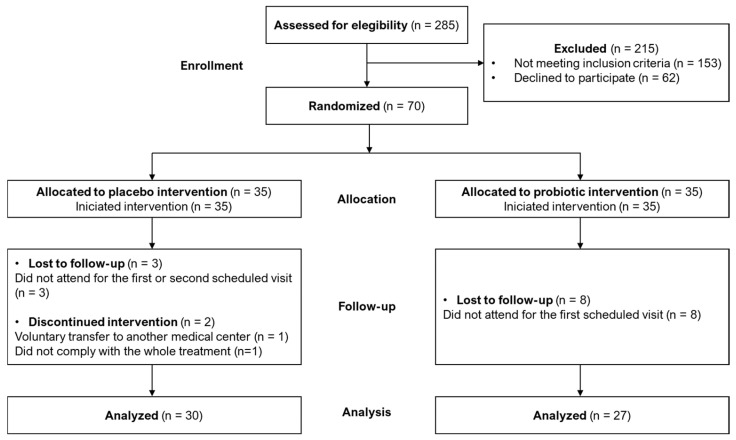
Flow diagram of distribution of participant couples during the trial according to CONSORT guidelines [27].

A total of 285 couples not being able to spontaneously conceive after 12 months of regular unprotected sexual intercourse who attended the Assisted Reproduction Service at the Hospital Clínico San Carlos (Madrid, Spain) were assessed for eligibility for this study (Figure 1). Among them, 153 couples did not meet the inclusion criteria, and 62 refused to participate in the study. Therefore, a total of 70 couples were randomized and allocated to the placebo (n = 35) or the probiotic (n = 35) groups and started the intervention. Among them, 5 couples in the placebo group and 8 couples in the probiotic group were lost to follow-up or discontinued the intervention. Finally, a total of 30 couples in the placebo group and 27 couples in the probiotic group completed the study, as indicated in Figure 1.

### 3.2. Characteristics of the Participants

Participants in the placebo group had a median (Q1, Q3) age of 36.52 (35.17, 37.88) years for females and 36.04 (34.53, 37.96) years for males, while in the probiotic group, the median (Q1, Q3) age was 37.02 (34.91, 38.29) years and 36.91 (33.40, 39.67) years for females and males, respectively. They did not differ in baseline age, weight, or height (Table 1). No significant differences in reproductive health (having children from another partner and/or abortions), infectious and autoimmune diseases, drug (antibiotics and corticosteroids) treatments, or other lifestyle or health-related habits were detected among males and females in both groups (Supplementary Tables S1 and S2). Alcohol consumption was highest among males in the probiotic group (81.5%) than in the placebo group (53.3%) (Chi-square test; *p* = 0.024; Supplementary Table S2), although this habit was mostly occasional in both groups. In contrast, no differences in alcohol consumption were observed among women in the placebo and probiotic groups (Supplementary Table S1).

### 3.3. Pregnancy Success and Other Clinical Outcomes

Characteristics of the IVF procedures and comparison of the clinical outcomes between the probiotic and the placebo groups are presented in Table 2. In both groups, there were three spontaneous pregnancies before ovarian stimulation (10.0% placebo vs. 11.1% probiotic group, *p* = 1.000).

After ovarian stimulation, 22 volunteers in the placebo group and 22 volunteers in the probiotic group underwent ovarian puncture (80.8% vs. 91.7%, Fisher’s exact test; *p* = 0.423) (Table 2). The duration of the intervention with the study products was similar between groups (mean days (95% CI): 154.9 (139.4–170.4) for placebo vs. 161.9 (145.8–178.0) for probiotic, one-way ANOVA; *p* = 0.659). Also, the time from ovarian stimulation to ovarian puncture was no different between groups (mean days (95% CI): 12.0 (11.0–13.0) for placebo vs. 11.9 (10.9–12.8) for probiotic, one-way ANOVA, *p* = 0.883).

Of those, 14 volunteers (66.7%) in the placebo group performed the embryo transfer, compared to 17 volunteers (77.3%) in the probiotic group (two-tailed Chi-square test; *p* = 0.322) (Table 2). The proportion of fresh versus frozen embryos transferred in cases resulting in pregnancy was similar between groups (Fisher’s exact test; *p* = 1.000). However, the pregnancy rate after the embryo transfer was significantly higher in the probiotic group (11 volunteers, 64.7%) compared to the placebo group (4 volunteers, 28.6%) (Fisher’s exact test; *p* = 0.045) (Table 2).

The rate of total pregnancies, including spontaneous and IVF-related pregnancies, as well as those ending in abortion, was 26.7% in the placebo group and 51.8% in the probiotic group (two-tailed Chi-square test; *p* = 0.051) (Table 2). However, a significant difference was observed in the number of successful pregnancies that resulted in a live baby without birth defects, the primary outcome of the study, between both groups: 6 out of 30 couples (20%) in the placebo group, and 13 out of 27 couples (48%) in the probiotic group (one-tailed Chi-square test; *p* = 0.024) (Table 2). The absolute risk increase (95% CI) in pregnancy rates for the probiotic group compared to the placebo group was 28 (5–52)%. These results indicate that probiotic supplementation was not inferior to the standard procedure (placebo) in achieving a successful pregnancy, as the lower limit of the 95% CI exceeded the pre-specified non-inferiority margin (10%). In addition, since the lower limit of the 95% CI was also greater than zero, these results suggest that probiotic treatment may be superior to the regular procedure (placebo).

During the intervention, and as reported by the participants up to the fourth visit, none of the couples experienced any side effects associated with the intake of the study product.

All pregnancies involved singleton fetuses, and all infants were born at term gestational age (37–42 weeks), except for one infant in the probiotic group, who was born at 34 weeks and 5 days. The cause of preterm birth was a premature rupture of membranes (PROM) at 34 + 5 weeks in the context of chorioamnionitis. More detailed maternal information, including pregnancy complications, the rate of group B streptococci (GBS)-positive women, delivery mode (vaginal or C-section), and infant data, including Apgar score, sex, birth weight and length, and neonatal complications, is provided in Supplementary Table S3. No statistically significant differences were found between the placebo and probiotic groups for any of these parameters (Supplementary Table S3).

### 3.4. Other Secondary Outcomes

#### 3.4.1. Microbial Characterization of Vaginal Exudates Using Culture-Dependent Methods

All women (n = 57) provided a vaginal exudate at recruitment (time 1), except one who did not provide any, neither at time point 1 or 2, although the couple completed the intervention. Among those couples that finished the trial, 11 failed to provide samples at time 2 (3 from the placebo group and 8 from the probiotic group; Chi-square test, *p* = 0.061). Therefore, a total of 101 samples of vaginal exudate were analyzed using culture-dependent methods (Supplementary Table S4). Microbial growth was observed in all of them, except in three vaginal exudates (two of them from one participant of the probiotic group).

In the vaginal exudate samples (n = 98), up to 442 distinct microbial isolates were obtained, the majority of which were bacteria (98%). Only nine yeast isolates were recovered, though they were not identified. Most of the bacterial isolates were assigned to the phyla *Bacillota* (73.5%) and *Actynomycetota* (23.1%), with the remaining isolates classified under *Pseudomonadota* (1.1%) and *Bacteroidota* (0.2%) (Supplementary Table S4). The bacterial isolates were classified into 36 different genera (Supplementary Table S4) and 101 different species. However, only 25 bacterial species were found in 5 or more samples, being *Lactobacillus crispatus*, *Staphylococcus epidermidis*, and *Enterococcus faecalis* the species most frequently found (in 58, 38, and 36 samples, respectively); half of the bacterial species (n = 51) were detected just in one sample only (Supplementary Figure S1).

The culture-dependent microbial diversity of the vaginal samples, as assessed using the Shannon and Simpson diversity indices, is presented in Supplementary Table S5. The median (Q1, Q3) number of distinct bacterial species identified in individual vaginal exudate samples was 4 (2, 6), ranging from 0 to 13. The mean (95% CI) bacterial count was 5.88 (5.63–6.13) log_10_ CFU/mL, with a range from 3.18 to 8.14 log_10_ CFU/mL. Samples collected at the end of the intervention exhibited a lower median number of distinct bacterial species and reduced bacterial counts compared to those collected at the beginning of the trial (Supplementary Table S5). No differences were found in the number of distinct bacterial species or in the Shannon and Simpson diversity indices between the placebo and probiotic groups at either sampling time. However, lower bacterial diversity indices (Shannon and Simpson) were observed in the vaginal samples collected at the end of the intervention from women who underwent embryo transfer and among women that achieved pregnancy, compared to the samples from women who did not achieve pregnancy during the trial (Supplementary Table S5).

No differences were found in the prevalence of any of the detected genera between the placebo and probiotic groups at both sampling times, including lactobacilli (both current and former members of the *Lactobacillus* genus) (Supplementary Table S6). However, the mean lactobacilli count in the placebo group decreased by 0.85 log_10_ CFU/mL at the end of the study, while no change was registered in the probiotic group (Supplementary Figure S2). Such a change in the *Lactobacillus* (*sensu stricto*) counts during the intervention period was statistically significant (one-way ANOVA; *p* = 0.020).

#### 3.4.2. Metataxonomic Analysis of Vaginal Exudates

Sequencing was performed on vaginal samples from 54 women at time point 1, generating a total of 22,485,985 reads, with a median of 273,720 sequences per sample, while at time point 2, only samples from 43 women were available, obtaining a total of 17,133,478 reads, with a median number of 277,263 sequences per sample. No differences were observed when comparing the median number of reads per sample of the placebo and the probiotic groups at the two sampling times, indicating consistent sequencing depth across both sampling times and treatment groups. Globally, the Shannon index values ranged from 0.01 to 1.86, while the Simpson index values ranged from <0.01 to 0.78. No significant differences were observed in the median values of the Shannon and Simpson indices between the samples taken before and after the intervention in either the placebo or probiotic groups (Wilcoxon rank-sum tests; *p* > 0.050 for Shannon and Simpson indices in both the placebo and probiotic groups) (Supplementary Figure S3A). Similarly, beta diversity analysis using Bray–Curtis and Jaccard distance matrices revealed no differences in microbial composition between the two intervention groups and time points, indicating no significant variations in the overall structure of the microbiota (Bray–Curtis: *p* = 0.971, Jaccard: *p* = 0.618) (Supplementary Figure S3B,C).

**Figure 2:**
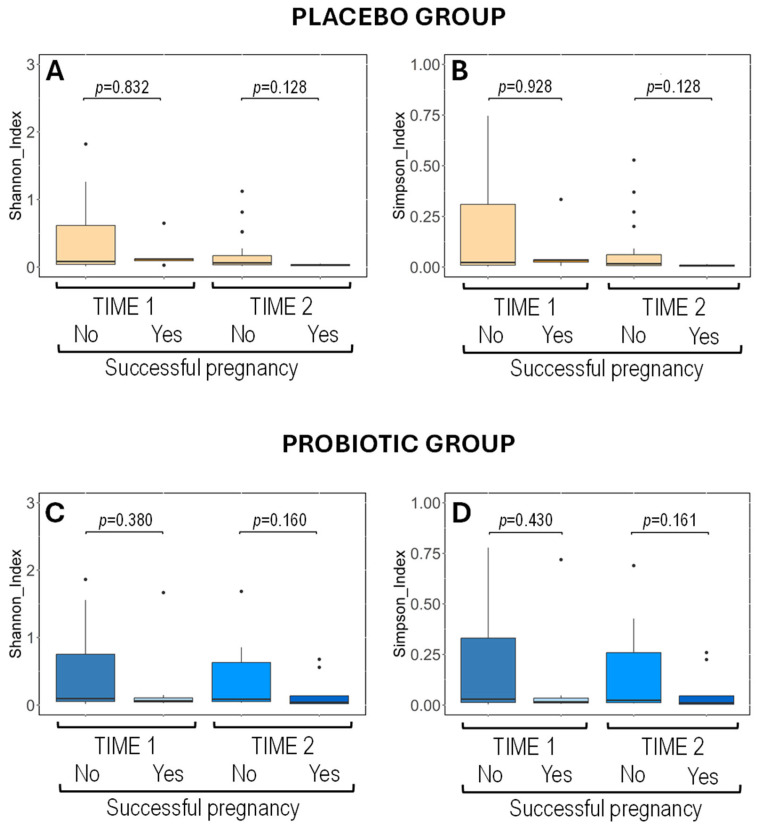
Metataxonomic analysis of vaginal exudates. Boxplots showing the alpha diversity of vaginal microbiota, measured by Shannon (**A**,**C**) and Simpson (**B**,**D**) indices, in women according to the intervention (placebo in upper graphs and probiotic in lower graphs), the sampling time point (Time 1, Time 2), and the primary outcome of the study (successful pregnancy or not). Wilcoxon rank-sum tests were used to compare the alpha diversity indices.

Wilcoxon rank-sum tests showed no significant differences in alpha diversity between the vaginal samples from women of the placebo and probiotic groups regardless of pregnancy outcome. These results indicate an evenness and consistent vaginal microbiota across treatment groups over time, independent of the primary outcome (Figure 2).

Metataxonomic profiling at the phylum level showed that *Firmicutes* (now *Bacillota*) dominated the vaginal microbiota in most vaginal samples, having median relative abundances close to 99% (Supplementary Figure S4, upper panel). Other phyla, including *Actinobacteriota*, *Proteobacteria* (now *Actinomycetota* and *Pseudomonadota*, respectively), *Fusobacteriota,* and *Bacteroidota,* were similarly present and distributed in the samples from the placebo and probiotic groups taken at sampling time points 1 and 2 (Supplementary Figure S4, upper panel).

At the genus level, *Lactobacillus* was the most abundant genus in the samples provided at both time points and from both treatment groups, and is present in 93% of the samples, having high relative abundances (≥95%) in 75% of the samples (Supplementary Figure S4, lower panel). Other genera, such as *Gardnerella*, *Bifidobacterium*, *Atopobium*, and *Anaerococcus*, were detected at lower frequencies and abundances. Pairwise comparisons using Wilcoxon rank-sum tests showed no significant differences in the relative abundances of these genera between the placebo and probiotic groups or between the two sampling time points (Supplementary Figure S4, lower panel).

Overall, the vaginal microbiota composition remained highly stable across the different groups and sampling points, with *Lactobacillus* dominating and only minor variations observed in the relative abundances of other taxa. *Megasphaera*, a minor genus, was less abundant in the samples from women at time point 1 who achieved pregnancy compared with those who failed to achieve pregnancy (*p* = 0.034). The genus *Gardnerella* was consistently present in women who achieved pregnancy and in women who failed to achieve pregnancy at both sampling time points 1 and 2, although always at a very low abundance.

#### 3.4.3. Detection of *L. salivarius* in Vaginal Exudates by RT qPCR Analysis

The detection and quantification of *L. salivarius* was assessed using alternative culture-independent methods, which offer greater sensitivity than classical culture methods, and sometimes even than metataxonomic analysis. The extracted DNA from the vaginal exudates was analyzed using specific primers for *L. salivarius* and the RT qPCR technique. Before the intervention, at time point 1, *L. salivarius* DNA was detected only in two samples (6.9%) from the placebo group and two samples (7.4%) from the probiotic group (Table 3).

Administration of *L. salivarius* CECT5713 led to a notable change in the detection and quantification of *L. salivarius* DNA. Specifically, there was a statistically significant difference between both groups regarding the number of samples in which *L. salivarius* DNA could be detected (Chi-square test, *p* < 0.001) (Table 3). It increased from 7.4% at time point 1 to 89.5% at time point 2 in the probiotic group while, in contrast, it could be detected only in two women from the placebo group (the same women that were positive at time 1), although the average concentration of *L. salivarius* DNA did not change (Table 3).

*L. salivarius* DNA concentration in the vaginal exudates were compared between women who achieved pregnancy and those having failed pregnancies, independently of the intervention group (probiotic or placebo) (Table 3). It was found that there was no difference in the percentage of positive *L. salivarius* DNA samples between both groups before the intervention. However, both the percentage of samples positive for *L. salivarius* DNA and the mean concentration of *L. salivarius* DNA were significantly higher in the samples from women who achieved pregnancy compared to those who did not (Chi-squared test; *p* = 0.019 for the percentage of positive samples, and Wilcoxon rank-sum test; *p* < 0.001 for *L. salivarius* DNA concentration) (Table 3). Similar results were obtained when considering the occurrence of successful pregnancies (Table 3).

**Figure 3:**
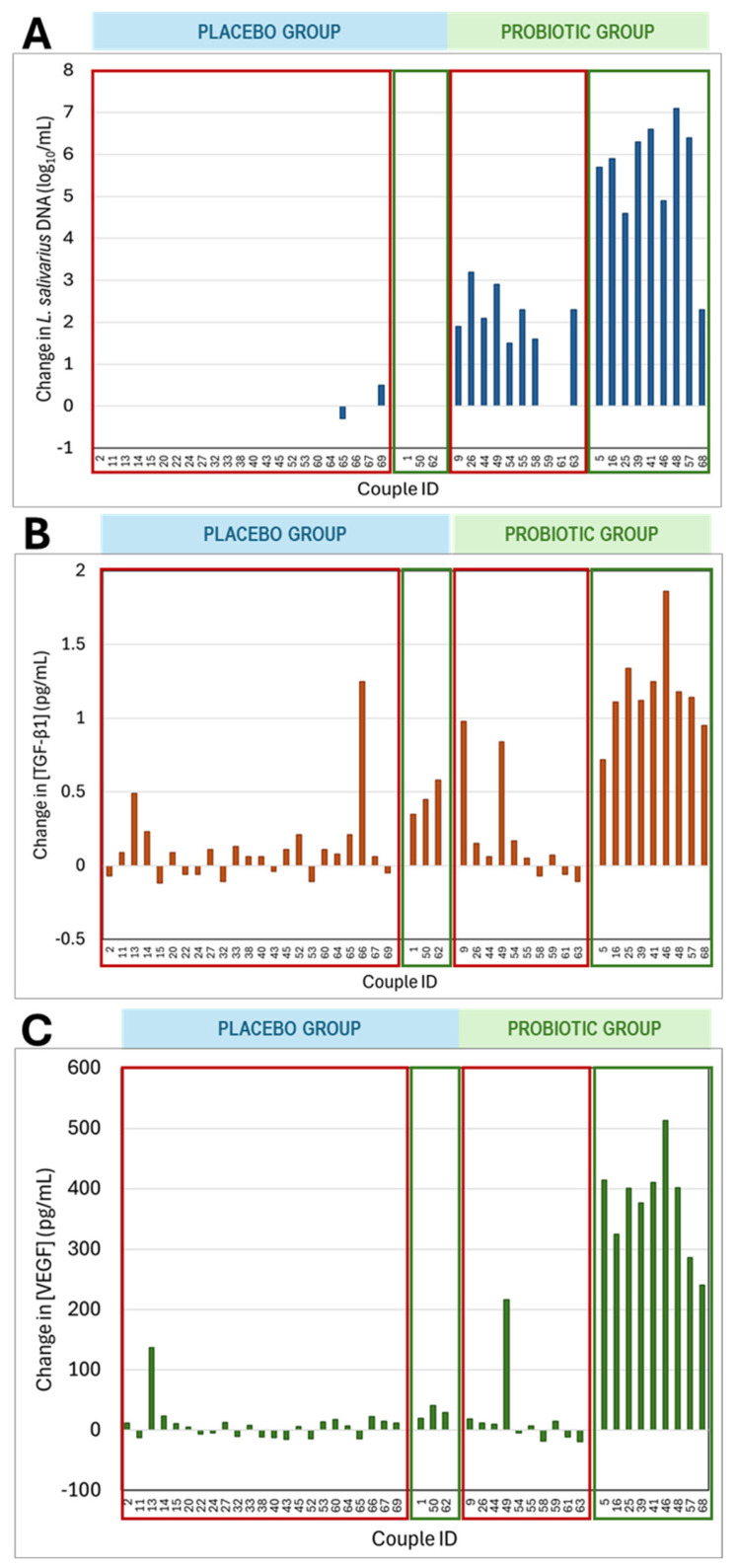
Change in the relative concentrations of (**A**) *L. salivarius* DNA (log_10_ CFU/mL) and the immunological parameters (**B**) TGFβ1 and (**C**) VEGF (pg/mL) in the vaginal exudate samples after the intervention in the placebo and probiotic groups. A red square was drawn around women who failed to achieve pregnancy or experienced miscarriage, and a green square indicates women with a successful pregnancy. Colored bars above the graphs indicate the treatment group (blue for placebo and green for probiotic).

Regarding longitudinal changes in *L. salivarius* DNA concentrations in vaginal exudates, the two *L. salivarius* DNA-positive samples of the placebo group showed similar DNA levels throughout the intervention period (Figure 3A). In contrast, after the intervention, the concentration of *L. salivarius* DNA in the probiotic group (detected in 90% of the samples) increased significantly, with the levels rising by 1.5 to 7.1 log_10_/mL copies compared to the baseline values (Figure 3A). All women in the probiotic group with term pregnancies showed a notable increase in *L. salivarius* DNA concentration, with a mean (95% CI) increase of 5.5 (4.4–6.6) log_10_/mL copies. In contrast, samples from women in the probiotic group with failed pregnancies showed only a mean (95% CI) increase of only 1.8 (1.0–2.6) log_10_/mL copies between the two sampling times, significantly lower than that observed in women with term pregnancies in the probiotic group (one-way ANOVA; *p* = 0.000) (Figure 3A).

#### 3.4.4. Immunological Characteristics of Vaginal Samples

The concentrations of TGFβ1 and VEGF in the vaginal exudates collected at time points 1 and 2 were also analyzed. At time 1, the concentration of TGFβ1 ranged from 0.82 to 1.98 pg/mL while the concentration of VEGF ranged from 66 to 279 pg/mL. A slightly, but statistically significant, higher concentration of TGFβ1 was observed in the samples from the probiotic group compared to those from the placebo group (Wilcoxon rank-sum test; *p* = 0.014) (Table 4). However, no significant difference was detected in the concentration of VEGF between these groups at time point 1 (Wilcoxon rank-sum test; *p* = 0.566) (Table 4).

At time point 2, the difference in TGFβ1 concentrations between samples from the probiotic and placebo was also significant, with higher levels in the probiotic group compared to the placebo (Wilcoxon rank-sum test; *p* = 0.002). Furthermore, administration of the probiotic led to notable changes in the mean VEGF concentration, which was approximately 2.5 times higher in the samples from the probiotic group compared to those from the placebo group (Wilcoxon rank-sum test; *p* = 0.019) (Table 4).

Additionally, the intervention resulted in an increase in the TGFβ1 concentration in the probiotic group but not in the placebo group (Wilcoxon signed-rank test; *p* = 0.020 and *p* = 0.453, respectively). Regarding VEGF, the intervention did not lead to a significant change in its concentration in the placebo group, although there was a trend toward an increase in the probiotic group (Wilcoxon signed-rank test; *p* = 0.926 and *p* = 0.058, respectively) (Table 4).

The TGFβ1 and VEGF concentrations in the vaginal exudates at time 1 and time 2 were compared between the women who experienced failure or achieved pregnancies, regardless of the intervention group (probiotic or placebo) (Table 4). The median concentrations of TGFβ1 and VEGF in vaginal exudates at time point 1 were higher in women who achieved pregnancy compared to those who did not (Wilcoxon rank-sum test; *p* < 0.001) (Table 4). These differences were even more pronounced at time point 2, with higher TGFβ1 and VEGF concentrations in the probiotic group compared to the placebo group (Wilcoxon rank-sum test; *p* < 0.001) (Table 4). Furthermore, the median concentration of TGFβ1 and VEGF was also significantly higher in women whose pregnancies resulted in a live birth (pregnancy success) compared to those who were not pregnant or suffered a miscarriage at both sampling times (Wilcoxon rank-sum test; *p* < 0.001) (Table 4).

Individual changes in TGFβ1 and VEGF concentrations in the vaginal exudates at the end of the intervention are presented in Figure 3B,C, according to their assignment to the probiotic or placebo groups and whether the pregnancy resulted in a live birth or not. In both the probiotic and placebo groups, some samples showed no change in TGFβ1 and VEGF concentrations, while others exhibited a marked increase. Vaginal samples from most women who had pregnancy success showed a significant increase in TGFβ1 and VEGF concentrations, whereas this increase was not observed in most women who did not achieve pregnancy or experienced pregnancy loss (Wilcoxon signed-rank test; *p * < 0.001 for both immunological compounds) (Figure 3B,C). In addition, the median (Q1, Q3) increase in TGFβ1 concentration was 0.45 (0.35, 0.58) pg/mL in samples from the three women with a live birth in the placebo group, while the increase was more than double (1.14 (1.11, 1.25) pg/mL) in the nine women from the probiotic group (Wilcoxon rank-sum test; *p* = 0.013) (Figure 3B). Similarly, for VEGF, the median (Q1, Q3) increase observed for the women with pregnancy success in the placebo group was only 29 (20, 41) pg/mL, whereas for the women from the probiotic group, it was more than 10-times higher (401 (325, 410) pg/mL) (Wilcoxon rank-sum test; *p* = 0.012) (Figure 3C).

#### 3.4.5. Microbial Characterization of Semen Samples Using Culture-Dependent Methods

Semen samples were provided by all participants at both sampling times with the same exceptions already mentioned for the women’s vaginal exudate samples. Therefore, a total of 101 samples of semen were available for microbial characterization using culture-dependent methods. Microbial growth was observed in all of them except in four semen samples (two of them from one participant of the placebo group).

In the semen samples where bacterial growth was observed (n = 97), a total of 479 distinct bacterial and two yeast isolates were recovered (Supplementary Table S4). All bacterial isolates belonged to either the phylum *Bacillota* (60.1%) or *Actinomycetota* (39.5%) and were classified into 27 different genera (Supplementary Table S4) and 95 different species. Similar to what was observed in the vaginal exudate samples, more than half of the bacterial species (n = 54) were isolated from one or two samples, while only 23 bacterial species were found in five or more different samples. *Staphylococcus epidermidis* and *Enterococcus faecalis* were the most frequently detected species, present in 46 and 36 samples, respectively. Most bacterial genera were also found in the vaginal exudate samples. Only ten genera were exclusively found in the semen samples, and, except for *Rothia*, fewer than five isolates were assigned to each of these minor genera (Supplementary Table S4).

The median (Q1, Q3) number of distinct bacterial species identified in individual semen samples was 5 (4, 6), ranging from 0 to 10 (Supplementary Table S7). The mean (95% CI) bacterial count was 4.15 (3.94–4.35) log_10_ CFU/mL, ranging from 1.70 to 6.70 log_10_ CFU/mL. No differences were observed between the samples collected at time points 1 and 2 regarding the number of distinct bacterial species and bacterial counts (Supplementary Table S7).

Subsequently, it was investigated as to whether these parameters, along with the Shannon and Simpson diversity indices, differed between the samples collected at time points 1 and 2 between intervention groups (placebo or probiotic), IVF procedures, pregnancy occurrence and type, and live birth success (Supplementary Table S7). No significant differences between the treatment groups were observed at any time point for any of the analyzed parameters. In the semen samples collected at time point 1, there was a trend toward a higher number of different bacterial species (Wilcoxon rank-sum test; *p* = 0.070) and bacterial counts (one-way ANOVA, *p* = 0.080) in couples who achieved pregnancy compared to those who did not (Supplementary Table S7). Among the couples who attained spontaneously pregnancy (n = 6), there was also a trend toward higher bacterial diversity according to Shannon and Simpson indices, compared to those who conceived after IVF (n = 15) (one-way ANOVA; *p* = 0.078; Wilcoxon rank-sum test; *p* = 0.080, respectively) in semen samples collected at time point 1 (Supplementary Table S7). Similarly, the semen samples collected at time point 1 from couples with successful pregnancies showed a trend toward higher Shannon index values (one-way ANOVA; *p* = 0.075) (Supplementary Table S7).

The bacterial count in the semen samples collected at time point 2 was significantly higher in couples who achieved a pregnancy compared to those who did not (one-way ANOVA, *p* = 0.032). No other significant differences in bacterial counts, number of distinct bacterial species, or Shannon and Simpson diversity indices were detected in the samples collected at time 2 between the placebo and probiotic groups, IVF procedure outcomes, pregnancy occurrence and type, and live birth success (Supplementary Table S7).

The profiles of the bacterial genera in the semen samples collected at time point 1 were similar in prevalence and abundance between participants in the placebo and probiotic groups (Supplementary Table S8). In addition, no changes in the semen bacterial profile after the intervention were observed (Supplementary Table S8).

#### 3.4.6. Metataxonomic Analysis of Semen Samples

A total of 90 semen samples were analyzed at points 1 and 2, generating approximately 60.6 million reads overall, with a median of about 670,000 reads per sample. The median number of reads per sample did not differ between the placebo and probiotic groups at either sampling time, suggesting a consisting sequencing depth across both time points and treatment groups, as it was also observed for vaginal exudate samples. In the entire dataset, the Shannon index values ranged from 0.45 to 3.20, and the Simpson index values ranged from 0.17 to 0.94. No significant differences were detected in the median values of the Shannon and Simpson indices between the samples collected before and after the intervention in the placebo group (Wilcoxon rank-sum tests; *p* > 0.050 for Shannon and Simpson indices) (Supplementary Figure S5A). In contrast, following the intervention in the probiotic group, the Simpson index showed a significant increase, whereas no change was observed in the Shannon index (Wilcoxon rank-sum tests; *p* = 0.010 and *p* = 0.127, respectively) (Supplementary Figure S5A). No differences in microbial composition were observed between the two intervention groups or across time points according to beta diversity analysis based on Bray–Curtis (*p* = 0.450) and Jaccard (*p* = 0.965) distance matrices, suggesting no substantial changes in the overall microbiota structure of the treatment groups after the intervention (Supplementary Figure S5B,C).

**Figure 4:**
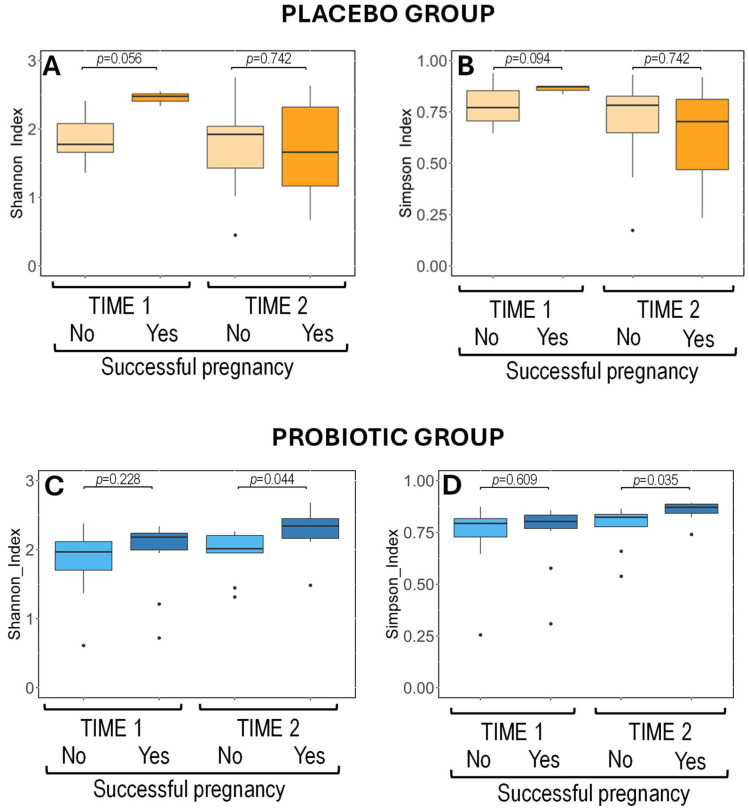
Metataxonomic analysis of semen samples. Boxplots showing the alpha diversity of semen microbiota, measured by Shannon (**A**,**C**) and Simpson (**B**,**D**) indices, according to the intervention (placebo in upper graphs and probiotic in lower graphs), the sampling time point (Time 1, Time 2), and the primary outcome of the study (successful pregnancy or not). Wilcoxon rank-sum tests with Bonferroni-corrected *p*-values were used to compare the alpha diversity indices among the four subgroups defined by the placebo and probiotic groups at both time points and are shown at the lower right corner of the graphs (statistical significance was considered at *p* < 0.05). Significant differences in alpha diversity of semen samples within the probiotic group after the intervention (time 2) between couples achieving successful pregnancy and those who did not are indicated in the upper right corner of the graphs for the probiotic group.

In the placebo group, semen samples from couples achieving pregnancy success (n = 3 for time 1 and n = 3 for time 2) and from those who did not achieve pregnancy or it was failed (n = 20 for time 1 and n = 21 for time 2) exhibited similar alpha diversity values at both time points (Wilcoxon rank-sum tests: *p* > 0.050; Figure 4) and relative abundance of the dominant bacterial phyla and genera at time point 1 (Supplementary Figure S6). These results indicate that the taxonomic profile and diversity were similar to the semen samples of the placebo group during the intervention.

The alpha diversity indices in the semen samples of the participants in the probiotic group collected before the intervention were comparable regardless of their final pregnancy outcome (n = 11 from successful pregnancies and n = 13 from men in couples who did not achieve pregnancy or experienced failed pregnancies) (Wilcoxon rank-sum tests; *p* = 0.228 and *p* = 0.609, for Shannon and Simpson indices, respectively) (Figure 4). However, after the intervention, significant differences emerged within the probiotic group based on reproductive outcomes (n = 9 from successful pregnancies and n = 10 samples from men in couples who did not achieve pregnancy or experienced failed pregnancies). Semen samples from the participants with successful pregnancies exhibited higher alpha diversity, with median (Q1, Q3) Shannon and Simpson indices of 2.37 (2.15, 2.46) and 0.88 (0.84, 0.89), respectively, compared to 2.06 (1.96, 2.24) and 0.82 (0.78, 0.84) for participants with unsuccessful pregnancies (Wilcoxon rank-sum tests; *p* = 0.044 and *p* = 0.035, for Shannon and Simpson indices, respectively) (Figure 4). These findings suggest improved community evenness over time in the semen samples from couples in the probiotic group achieving pregnancy success.

Detailed metataxonomic profiling identified the key bacterial phyla present in all semen samples, including *Firmicutes* (the most abundant; now *Bacillota*), *Actinobacteriota* (now *Actinomycetota*), *Bacterioidota*, *Proteobacteria* (now *Pseudomonadota*), and *Fusobacteroidota*. Additionally, the most abundant genera were *Corynebacterium*, *Staphylococcus*, and *Streptococcus* (Supplementary Figure S6). No metataxonomic differences were observed in the microbial community composition at either the phylum or genus level in the semen samples from the probiotic group collected before and after the intervention (Supplementary Figure S6).

#### 3.4.7. Immunological Characteristics of Semen Samples

The immunological analysis revealed the presence of the following immunological compounds in all available semen samples: IL-2, IL-4, IL-6, IL-8, IL-10, GM-CSF, IFNγ, TNFα, and TFGβ1 (Table 5). In the samples collected before the intervention, no significant differences in compound concentrations were observed between the study groups (placebo or probiotic), except for IL-4 and TNFα, the concentrations of which were slightly higher in the semen samples from the probiotic group compared to the placebo group. In contrast, after the intervention, statistically significant differences were found in the concentrations of these compounds, as well as for IL-6, IFNγ, and TFGβ1, between the placebo and probiotic groups. For all compounds except TFGβ1, the concentrations were lower in the probiotic group. Notably, the TFGβ1 level was 142% higher in the probiotic group (Wilcoxon rank-sum test; *p* < 0.002 for IL-2 and *p* < 0.001 for the remaining compounds) (Table 5).

When comparing the concentrations of these cytokines in the semen samples based on primary (successful pregnancy vs. not pregnancy plus miscarriages) and secondary (pregnancy vs. not pregnancy) outcomes, no significant differences were observed in the samples collected at time point 1 except for IL-6, which was lower in the samples from couples who attained pregnancy during the study. However, in the semen samples collected after the intervention, the concentrations of IL-4, IL-6, IL-8, IFNγ, TNFα, and TFGβ1 differed between the samples from couples that achieved pregnancy and those who did not (Table 5).

To gain further insight into the probiotic effect on the immunological profile of the semen, changes in the concentrations of these compounds in the participants between the time points were analyzed according to the intervention group. None of the immunological compounds changed significantly after the intervention in the semen samples from the placebo group. In contrast, significant changes were observed in the probiotic group at the end of the intervention for more than half of the measured cytokines. The concentrations of IL-6, IFNγ, and TNFα decreased by approximately 50%, and IL-4 by 19%. The most notable change was observed for the TFGβ1 concentration, which nearly doubled from a mean (95% CI) of 257.3 (145.8, 361.3) ng/mL at time point 1 to 491.8 (431.8, 573.0) ng/mL at the end of the intervention (Table 5). Similar results were obtained when considering the occurrence or non-occurrence of successful pregnancies (Table 5).

## 4. Discussion

In this randomized controlled clinical study, oral administration of *L. salivarius* CECT5713 to couples diagnosed as having unexplained infertility and scheduled for IVF led to a significant increase in the successful pregnancy rate in comparison to the placebo group, confirming the findings of a previous no-placebo-controlled open-labeled trial involving the administration of the same strain to women with reproductive failure (repetitive abortion or unexplained infertility) [17].

In this work, the analysis of vaginal exudates using culture-dependent methods showed that the mean lactobacilli count in the placebo group decreased from the beginning to the end of the study, while no change was registered in the probiotic group. In addition, there was a lower bacterial diversity in the vaginal samples collected at the end of the intervention from women that achieved pregnancy in comparison compared to those obtained from women who did not achieve pregnancy during the trial. Previously, it has been reported that deviations from a low-diversity and *Lactobacillus*-rich composition of the vaginal microbiota may have a negative impact on fertility and in the outcomes of assisted-reproduction procedures [17,28,29,30]. It has also been suggested that the decrease in the overall diversity of the vaginal microbiota is a better indicator of the risk of negative pregnancy outcomes than the presence or absence of specific bacterial species [31].

The metataxonomic analysis of the DNA extracted from the vaginal samples did not reveal a significant change in the composition of the vaginal bacteriome after the probiotic treatment. However, this kind of analysis frequently fails at finding differences when assessing microbiomes characterized by a high dominance of a given genus, such as happens in the vaginal ecosystem in relation to the genus *Lactobacillus*. Partial amplification and sequencing of the 16S rDNA gene allows for the comparison of sequences at the genus level but not at the species or strain level. Consequently, the fact that *Lactobacillus* sequences accounted for a very high proportion of the total sequences at both sampling times in most of the women recruited in our study precluded the finding of differences in the microbiome associated with probiotic intake. Similarly, no changes or very minor ones have been observed in the vaginal microbiomes of healthy women after being treated with probiotics [32,33,34]. A trial comparing the oral administration of a probiotic product or a placebo to asymptomatic pregnant women found that the vaginal microbiomes of both groups were similar, both regarding diversity and abundance, at the end of the 12-week treatment and also at week 35 of gestation [35]. Although in vitro studies had predicted that one of the strains in the probiotic product (*Lacticaseibacillus rhamnosus* GR-1) would have the ability to alter the vaginal microbiome after oral administration, the trial showed that this was not the case in practice [35]. It must be noted that women recruited in this trial had unexplained infertility but were otherwise healthy and had no vaginal infections.

In contrast, in cases of clinical vulvo-vaginal infections, the etiological agent may account for a relatively high proportion of sequences, and if a probiotic strain is able to displace it, then a shift in the microbiome profile may occur, as described by Vujic et al. [36] in a probiotic trial involving women with bacterial vaginosis. Similar results were found in another trial in which women with the same condition were treated with a probiotic as an adjunct therapy to treat the infection, while the vaginal bacteriome remained unchanged when the same probiotic preparation was used for the treatment of vulvovaginal candidiasis [37].

In contrast to the metataxonomic approach, analysis of the DNA samples using a *L. salivarius* species-specific PCR assay showed that this species was present in the vaginal ecosystem of the women of the probiotic group after the intervention, suggesting that a modification of the vaginal microbiota occurred, at least transitorily, at the strain level. Such moderate but relevant increases in the *L. salivarius* DNA load in the vaginal samples of the probiotic group remain unnoticed in the metataxonomic analysis since the autochthonous vaginal species of each woman were still present at much higher densities after the treatment, and all of them (*L. salivarius* and autochthonous lactobacilli DNA) were retrieved as *Lactobacillus* sequences by the bioinformatics analysis of the raw data.

So far, microbiome assessments during probiotic trials have usually been performed to try to explain their efficacy for preventing or treating a condition through modulation of the microbiome, but most of them have failed to report a relevant impact on the local microbiota [38]. Interestingly, it has been recently suggested that probiotic-related microbiome shifts may be regarded as a potential safety concern, and therefore, such analysis should be included as a part of the safety assessment of a probiotic product [39]. The potential risks include a negative impact on the structure and function of the host microbiota or the displacement of a microbe that may play a key role in local or general function and health [39]. The pertinence of such an additional safety assessment has been stressed after mice and human studies revealed that certain multi-strain probiotic products administered during or after antibiotic treatment delayed the recovery of the gut microbiota composition and function for at least 6 months in comparison with the control or placebo groups [40,41,42]. It has been postulated that such probiotic-related microbiota alterations may predispose or be a risk factor for infectious, inflammatory, and metabolic conditions later in life [43].

In this frame, it has been suggested that the actual impact of probiotics does not reside in their potential to modify the host’s target microbiota but rather in their ability for a direct interaction with epithelial and immune cells by sharing gene products and metabolites [38]. This study found that oral administration of *L. salivarius* CECT5713 to women with infertility of unknown origin is associated to an increase in the vaginal concentrations of VEGF and TGFβ1, which is in agreement with the results obtained with the same strain and with other *L. salivarius* strains in previous pilot open-labelled trials targeting the same condition and, also, women with repetitive abortion [17,44]. As previously discussed in these two studies, changes in the levels of such growth factors are particularly relevant for the primary outcome of the trial. Increased expression of VEGF, TGFβ1, and their respective receptors seems critical for embryogenesis and embryo implantation, and for a proper vascular function of the endometrium. As a result, any impairment in their levels during early pregnancy may lead to an unsuccessful implantation or abortion during the first trimester of pregnancy [45,46]. Both factors have complementary and synergistic functions, and their expression is highly regulated and coordinated during the earliest stages of pregnancy [47], suggesting a link between the action of both growth factors. In addition, TGFβ1 participates very actively in the induction of active immune tolerance in mucosal tissues, promoting Treg responses and maternal immune tolerance towards the embryo and the subsequent fetus [48]. Precisely, decreases in local Treg and tolerogenic immune cells increase the risk of pregnancy loss [49,50].

In this work, the treatment of male partners with *L. salivarius* CECT5713 led to a significant decrease in the seminal concentrations of IL-4, IL-6, IFNγ, and TNFα, and a significant increase in those of GM-CSF and TGFβ1, with respect to the basal levels. In contrast, opposite changes were observed for these immunological parameters in the placebo group. Seminal cytokines seem to play relevant roles in male reproductive health [51]. In fact, COVID-19-associated male infertility may involve a decrease in testosterone levels and spermatogenesis through shifts in cytokines such as IL-4, IL-6, IFNγ, and TNFα [52]. Some studies have reported that seminal levels of IL-6 are significantly higher in infertile men when compared to fertile men [53,54], while others have failed to find such a relationship [55]. Conflicting results have also been found regarding TNFα, from a negative correlation with sperm motility and morphology [55] to no relationship with semen quality or parameters of sperm functional capacity in asymptomatic infertile individuals [54]. Eggert-Kruse et al. [56] reported that TNFα levels in seminal plasma correlated with leukocyte counts, suggesting a silent male genital tract inflammation, but these authors did not find any association between the TNFα concentration and clinically relevant parameters of semen quality. More recently, it has been observed that TNFα-specific microRNA was more expressed in the seminal plasma of oligozoospermic patients than in that from controls, but no correlation between this finding and sperm parameters could be established [57].

The increase in GM-CSF levels in seminal plasma after probiotic intake seems particularly relevant, since it has been repeatedly observed that this cytokine improves sperm quality in vitro [58,59,60,61] and embryo development and implantation in animal models [62]. Finally, mouse studies have found that the elevated level of TGFβ usually present in semen is necessary for boosting uterine Treg cells after coitus and prior to fertilization and embryo implantation [63,64]. Seminal TGFβ needs to be activated to become functional [65,66], and such activation is mainly driven by an acidic environment pH [63]. Interestingly, it has been previously found that oral administration of *L. salivarius* CECT5713 led to an increase of the vaginal levels of TGFβ1 and, simultaneously, to a notable decrease in the vaginal pH values [17]

The relevance of a proper and thoughtful selection and characterization of any strain aimed at increasing fertility rates is highlighted by the fact that other strains have failed to improve pregnancy rates [67,68,69]. It is interesting to note that, in all of these trials reporting no relevant effect, the probiotic product was administered vaginally while the oral route was used in another trial that showed a positive impact of the probiotic strain in fertility outcomes [70]. In our case, the probiotic strain was administered orally, too. In fact, the lack of fecal samples may be considered a limitation of this trial, since it is possible that the metabolic and immunomodulatory properties of the strain in the human gut may play relevant roles in increasing pregnancy rates. The gut mucosa and the vaginal mucosa are integrated in the mucosal-associated lymph tissue system, and therefore, modulation of gut metabolism and immune function may have a reflect in the female genitourinary system [71,72,73]. As an example, estrogens are pivotal for human reproductive physiology through a wide variety of pleiotropic effects on both reproductive and non-reproductive organs. In this frame, the study of the repertoire of genes within the gut microbiota that are able to metabolize estrogens (the so-called estrobolome [74]) has gained great interest in the last years [75]. Recently, it has been described that *L. salivarius* strains may affect the fate of endogenous estrogens, including the degradation and conjugation of 17β-estradiol, the most potent estrogenic compound [76].

Another limitation faced by this study was the fact that the start of the trial overlapped with the COVID-19 pandemic. As a result, some of the final samples that should have been collected from couples who finished the trial were not available. Unfortunately, most of such lacking samples corresponded to women who achieved a term pregnancy and who had been randomly allocated to the probiotic group (31% of the samples that should have been collected from this group at time 2). It is possible that differences between both study groups might have been more pronounced if such samples had been available. Finally, this study was designed as a non-inferiority trial rather than a superiority trial to evaluate the efficacy of probiotic supplementation before IVF compared to the standard procedure to achieve a successful pregnancy. This decision was based on the consideration that the probiotic might offer only a modest benefit, which would necessitate an impractically large trial to demonstrate superiority. Therefore, the number of participating couples was relatively low, limiting the statistical power.

The results obtained in this study are highly promising. However, they should be considered preliminary, given the relatively small sample size, even though it was sufficient to support statistical significance. These findings also underscore that unexplained infertility is not a homogeneous condition, as not all couples responded to the probiotic treatment. Future studies with larger sample sizes are advisable to better elucidate the mechanisms underlying the observed probiotic success and to identify specific biomarkers that could guide more personalized and optimal treatment protocols. Larger trials would also allow for the identification of specific microbiological profiles in both vaginal exudates and semen, as well as probiotic-induced modifications that may explain the changes in bacterial diversity observed in this study. Additionally, a metabolomic analysis of vaginal exudates and semen could help determine whether other molecules present in the vaginal environment favor the interaction between the oocyte and sperm, and/or the implantation of the fertilized egg. In any case, as infertility rates continue to rise, affecting millions of couples worldwide, the effectiveness of the administration of the probiotic *L. salivarius* CECT5713 represents a cost-effective intervention that could substantially improve the outcomes of FIV procedures and may be feasible even in lower-resource settings.

## 5. Conclusions

Oral administration of *L. salivarius* CECT5713 significantly increased the successful pregnancy rate among couples with unexplained infertility. This outcome might be related to the ability of the probiotic strain to increase the vaginal concentration of the growth factors VEGF and TGFβ1 in women, without modifying the microbiota environment, and to the improvement in the semen quality in men. These results add evidence to the previous clinical data [17] that corroborate the potential of *L. salivarius* CECT5713 to enhance the fertility outcomes in couples with infertility of an unknown origin.

## Figures and Tables

**Table 1:** Baseline demographic characteristics, gynecological history, and reproductive data of participant couples.

Demographic Characteristics	Placebo Group (N = 30) Median (Q1, Q3)	Probiotic Group (N = 27) Median (Q1, Q3)	*p*-Value ^1^
Women			
Age at recruitment (years)	36.52 (35.17, 37.88)	37.02 (34.91, 38.29)	0.533
Weight (kg)	61.5 (56.0, 71.0)	60.0 (56.0, 67.0)	0.455
Height (cm)	162.5 (158.0, 168.0)	163.0 (158.0, 167.0)	0.867
Male partners			
Age at recruitment (years)	36.04 (34.53, 37.96)	36.91 (33.40, 39.67)	0.835
Weight (kg)	80.0 (74.0, 90.0)	77.0 (75.0, 85.0)	0.437
Height (cm)	178.5 (175.0, 183.0)	178.0 (173.0, 181.0)	0.423

N, total number of participants in the group. ^1^ Differences between groups (placebo, probiotic) were tested using the Wilcoxon rank-sum test.

**Table 2:** Details of the IVF procedures and clinical outcomes during intervention.

	Placebo Group (N = 30) n/N (%)	Probiotic Group (N = 27) n/N (%)	*p*-Value ^1^
Spontaneous pregnancies before ovarian stimulation	3/30 (10.0)	3/27 (11.1)	1.000
Ovarian puncture			
No	5/27 (19.2)	2/24 (8.3)	0.423
Yes	22/27 (80.8)	22/24 (91.7)	
Embryo transfer			
No	8/22 (33.3)	5/22 (22.7)	0.322 *
Yes	14/22 (66.7)	17/22 (77.3)	
Frozen embryo	10/14 (71.4)	13/17 (76.5)	1.000
Fresh embryo	4/14 (28.6)	4/17 (23.5)	
Pregnancy after embryo transfer			
No	10/14 (71.4)	6/17 (35.3)	0.045 *
Yes	4/14 (28.6)	11/17 (64.7)	
Frozen embryo	3/4 (75.0)	9/11 (81.8)	1.000
Fresh embryo	1/4 (25.0)	2/11 (18.2)	
Total pregnancies ^2^	8/30 (26.7)	14/27 (51.8)	0.051 *
Abortions	2/30 (6.7)	1/27 (3.7)	0.540
Successful pregnancies	6/30 (20.0)	13/27 (48.1)	0.024 *

N, total number of participants in the group; n, number of participants with the specific characteristic described. ^1^ Two-tailed Fisher’s exact tests and Chi-square tests (marked with an asterisk) were used to compare categorical variables between the placebo and probiotic groups. For the primary outcome (successful pregnancies), a one-tailed Chi-square test was performed at a significant level of 0.025 to compare pregnancy rates that resulted in a live baby without birth defects (successful pregnancy) between the placebo and probiotic groups. ^2^ Total pregnancies include both spontaneous (before and after ovarian stimulation) and IVF-assisted pregnancies.

**Table 3:** Prevalence (%) and concentration (log_10_ copies/mL) of *Ligilactobacillus salivarius* DNA in vaginal exudates according to the intervention group and the main outcomes of the study.

	Time 1		*p*-Value ^1^	Time 2		*p*-Value
	Placebo group	Probiotic group		Placebo group	Probiotic group	
Prevalence, n/N (%) ^2^	2/29 (6.9)	2/27 (7.4)	1.000	2/26 (7.7)	17/19 (89.5) ^#^	<0.001 *
Concentration, median (Q1, Q3)	2.15 (1.60, 2.70)	2.10 (1.80, 2.40)	0.699	2.25 (2.10, 2.40)	4.60 (2.30, 5.90)	0.352
	Not pregnancy	Pregnancy		Not pregnancy	Pregnancy	
Prevalence, n/N (%)	2/35 (5.7)	2/21 (9.5)	1.000	9/30 (30.0) ^#^	10/15 (66.7) ^#^	0.019 *
Concentration, median (Q1, Q3)	2.15 (1.60, 2.70)	2.10 (1.80, 2.40)	0.699	2.10 (1.90, 2.30)	5.80 (4.70, 6.40) ^#^	<0.001
	Not pregnancy + miscarriage	Successful pregnancy		Not pregnancy + miscarriage	Successful pregnancy	
Prevalence, n/N (%)	2/38 (5.3)	2/18 (11.1)	0.589	10/35 (30.0) ^#^	9/12 (66.7) ^#^	0.007
Concentration, median (Q1, Q3)	2.15 (1.60, 2.70)	2.10 (1.80, 2.40)	0.699	2.20 (1.90, 2.40)	5.90 (4.90, 6.40) ^#^	<0.001

N, total number of participants in the group; n, number of positive samples for the presence of *L. salivarius* DNA within the intervention or outcome group. ^1^ Fisher’s exact tests or Chi-square tests (marked with an asterisk) were used to compare categorical variables between groups or outcomes, while Wilcoxon rank-sum tests were used for continuous variables. Hash symbol (#) indicates statistically significant differences (*p* < 0.050) between time points within each intervention group (placebo, probiotic) or outcome group (“not pregnancy”, “pregnancy”, “not pregnancy + miscarriage”, “successful pregnancy”). ^2^ Prevalence was calculated as the number of positive samples for the presence of *L. salivarius* DNA out of the total samples available for each group or outcome.

**Table 4:** Concentration (pg/mL) of TGFβ1 and VEGF in vaginal exudates at both time points, stratified by intervention group and study outcomes.

	Time 1		*p*-Value ^1^	Time 2		*p*-Value
	Placebo group (n = 29)	Probiotic group (n = 27)		Placebo (n = 26)	Probiotic (n = 19)	
TGFβ1 ^2^	1.29 (1.11, 1.44)	1.42 (1.25, 1.72)	0.014	1.25 (1.17, 1.62)	2.33 (1.27, 2.97) ^#^	0.002
VEGF	119.0 (98.0, 154.0)	133.0 (98.0, 150.0)	0.566	110.0 (97.0, 140.0)	349.0 (101.0, 529.0)	0.019
	Not pregnancy (n = 35)	Pregnancy (n = 21)		Not pregnancy (n = 30)	Pregnancy (n = 15)	
TGFβ1	1.25 (1.06, 1.37)	1.67 (1.49, 1.83)	<0.001	1.23 (1.17, 1.42)	2.69 (2.27, 3.01) ^#^	<0.001
VEGF	102.0 (85.0, 122.0)	162.0 (145.0, 217.0)	<0.001	107.5 (92.0, 123.0)	431.0 (303.0, 564.0) ^#^	<0.001
	Not pregnancy + miscarriage (n = 38)	Successful pregnancy (n = 18)		Not pregnancy + miscarriage (n = 33)	Successful pregnancy (n = 12)	
TGFβ1	1.27 (1.07, 1.38)	1.72 (1.57, 1.86)	<0.001	1.27 (1.19, 1.45)	2.86 (2.38, 3.04) ^#^	<0.001
VEGF	104.0 (87.0, 130.0)	160.0 (145.0, 231.0)	<0.001	109.0 (94.0, 129.0)	485.5 (347.0, 582.5) ^#^	<0.001

n, number of samples analyzed within each intervention or outcome group. ^1^ Wilcoxon rank-sum tests were used to compare continuous variables between groups or outcomes and between both sampling times in the different groups or outcomes. Hash symbol (#) indicates significant differences (*p* < 0.050) between time points within each intervention group (placebo, probiotic) or outcome group (“not pregnancy”, “pregnancy”, “not pregnancy + miscarriage”, “successful pregnancy”). ^2^ Concentration of immunological compounds are expressed as median (Q1, Q3).

**Table 5:** Immunological profile of semen samples according to the intervention group and the main outcomes of the study.

Compound ^2^	Time 1		*p*-Value ^1^	Time 2		*p*-Value
	Placebo group (n = 29)	Probiotic group (n = 27)		Placebo group (n = 25)	Probiotic group (n = 19)	
IL-2	3.56 (3.41, 3.82)	3.59 (3.31, 3.87)	0.646	3.67 (3.45, 3.99)	3.47 (3.23, 3.71)	0.090
IL-4	0.58 (0.53, 0.63)	0.65 (0.60, 0.69)	<0.001	0.61 (0.57, 0.66)	0.53 (0.43, 0.61) ^#^	0.002
IL-6	3.27 (2.74, 3.71)	3.62 (3.23, 3.82)	0.103	3.41 (3.12, 3.76)	1.88 (1.43, 2.72) ^#^	<0.001
IL-8	197.5 (141.2, 298.5)	247.7 (201.9, 325.5)	0.156	201.8 (164.6, 277.8)	200.1 (177.4, 311.6)	0.434
IL-10	2.45 (2.12, 2.90)	2.34 (1.93, 3.06)	0.731	2.45 (2.14, 2.92)	2.48 (2.03, 2.84)	0.653
GM-CSF	1.29 (1.17, 1.42)	1.31 (1.17, 1.45)	0.889	1.26 (1.14, 1.49)	1.34 (1.23, 1.47)	0.192
IFNγ	50.94 (40.11, 64.60)	52.03 (44.94, 62.08)	0.664	48.81 (40.23, 69.66)	27.17 (21.29, 33.07) ^#^	<0.001
TNFα	6.64 (4.94, 7.03)	7.14 (6.50, 7.87)	0.022	6.17 (5.18, 7.23)	4.04 (3.87, 5.02) ^#^	<0.001
TGFβ1	348.2 (245.3, 398.6)	257.3 (145.8, 361.3)	0.091	345.5 (234.7, 388.1)	491.8 (431.8, 573.0) ^#^	<0.001
	Not pregnancy (n = 35)	Pregnancy (n = 21)		Not pregnancy (n = 30)	Pregnancy (n = 14)	
IL-2	3.59 (3.29, 4.02)	3.53 (3.41, 3.78)	0.987	3.65 (3.33, 3.96)	3.62 (3.44, 3.71)	0.830
IL-4	0.62 (0.58, 0.67)	0.59 (0.56, 0.67)	0.351	0.61 (0.57, 0.65)	0.45 (0.43, 0.48) ^#^	<0.001
IL-6	3.59 (3.23, 3.87)	3.33 (1.99, 3.69)	0.043	3.29 (3.02, 3.71)	1.53 (1.41, 1.95) ^#^	<0.001
IL-8	237.1 (178.4, 325.5)	207.1 (134.8, 265.7)	0.126	228.9 (180.2, 301.4)	183.7 (142.6, 200.1)	0.031
IL-10	2.54 (2.01, 3.07)	2.32 (1.91, 2.74)	0.150	2.59 (2.10, 2.92)	2.48 (2.06, 2.65)	0.512
GM-CSF	1.31 (1.17, 1.46)	1.28 (1.15, 1.38)	0.630	1.27 (1.14, 1.42)	1.36 (1.23, 1.59)	0.107
IFNγ	58.10 (41.40, 69.69)	47.48 (44.21, 54.29)	0.148	48.67 (35.12, 67.02)	23.47 (21.19, 29.12) ^#^	<0.001
TNFα	6.91 (5.73, 7.93)	6.71 (4.97, 7.26)	0.356	6.03 (5.02, 7.23)	4.02 (3.87, 4.82) ^#^	<0.001
TGFβ1	347.3 (164.2, 402.1)	257.3 (240.1, 333.9)	0.498	374.4 (245.7, 451.8)	489.9 (415.2, 579.3) ^#^	0.004
	Not pregnancy + miscarriage (n = 38)	Successful pregnancy (n = 18)		Not pregnancy + miscarriage (n = 33)	Successful pregnancy (n = 11)	
IL-2	3.58 (3.31, 3.87)	3.56 (3.38, 3.78)	0.958	3.65 (3.33, 3.94)	3.62 (3.44, 3.71)	0.968
IL-4	0.62 (0.58, 0.67)	0.59 (0.49, 0.67)	0.312	0.61 (0.57, 0.64)	0.44 (0.41, 0.46) ^#^	<0.001
IL-6	3.47 (3.21, 3.87)	3.42 (1.99, 3.71)	0.188	3.24 (2.98, 3.58)	1.46 (1.27, 1.64) ^#^	<0.001
IL-8	232.6 (178.3, 310.2)	223.4 (134.8, 278.4)	0.366	221.8 (178.4, 297.1)	199.0 (134.5, 261.5)	0.175
IL-10	2.46 (2.01, 3.06)	2.26 (1.91, 2.79)	0.258	2.45 (2.10, 2.92)	2.48 (2.06, 2.69)	0.892
GM-CSF	1.32 (1.18, 1.48)	1.28 (1.09, 1.35)	0.247	1.27 (1.15, 1.45)	1.34 (1.23, 1.59)	0.350
IFNγ	55.67 (40.11, 69.69)	48.27 (45.42, 54.29)	0.277	48.52 (35.17, 67.02)	22.38 (20.29, 29.12) ^#^	<0.001
TNFα	6.91 (5.67, 7.93)	6.80 (5.29, 7.26)	0.516	5.92 (4.98, 7.14)	3.94 (3.52, 4.04) ^#^	<0.001
TGFβ1	347.7 (174.5, 402.1)	274.2 (229.2, 333.2)	0.273	382.4 (299.7, 444.3)	503.1 (486.5, 594.3) ^#^	0.002

n, number of samples analyzed within each intervention or outcome group. IL, interleukin; GM-CSF, granulocyte macrophage colony-stimulating factor; IFNγ, interferon γ; TNFα, tumor necrosis factor α; TGFβ1, transforming growth factor β1. ^1^ Wilcoxon rank-sum tests were used to compare the concentrations of immunological compounds between groups or outcomes. Hash symbol (#) indicates statistically significant differences (*p* < 0.001) in median concentrations between time points within each intervention group (placebo, probiotic) or outcome group (“not pregnancy”, “pregnancy”, “not pregnancy + miscarriage”, “successful pregnancy”). ^2^ Concentration of immunological compounds are expressed as median (Q1, Q3) and in pg/mL except for TGFβ1 that it is expressed as ng/mL.

## Data Availability

The rest of the data presented in this study are available upon request from the corresponding author. Data are not public due to ethical reasons.

## Supplementary Materials

The following supporting information can be downloaded at https://www.mdpi.com/article/10.3390/nu17111860/s1: Figure S1: Stacked bar chart depicting the relative abundance of the main bacterial genera (present in ≥5 samples) in vaginal exudate samples from the placebo (n = 29; one participant did not provide any sample) and probiotic (n = 27) groups, assessed using classical culture techniques. Colored bars above the graph indicate the group assignment (placebo or probiotic group) and the study outcomes (successful pregnancy resulting in live birth pregnancy or no successful pregnancy). For pregnancies, the mode of conception is further specified as either spontaneous [SPONT] or after an IVF procedure [IVF]. Each participant woman has two stacked bars: the first corresponds to the first sampling point (T1, blue dot) and the second to the second sampling point (T2, green dot). Figure S2: Box plots showing the abundance of lactobacilli (including current and former members of the *Lactobacillus* genus) in vaginal exudate samples from the placebo and probiotic groups at both sampling points (T1 and T2), assessed using classical culture techniques. No significant differences in the prevalence of lactobacilli (≥85%) were found between the placebo and probiotic groups, as determined by Chi-square tests. One-way ANOVA was used to compare samples collected at sampling time 1 (T1) and 2 (T2) within the placebo and the probiotic group separately. Figure S3: A: Boxplots showing the alpha diversity (Shannon and Simpson indices) of vaginal microbiota in women from the placebo (orange) and probiotic (blue) groups, at sampling time points 1 (light color) and 2 (dark color). Wilcoxon rank-sum tests were used to compare the change in alpha diversity indices before and after the intervention in the placebo and probiotic groups. B and C: Beta diversity (PCoA) of vaginal microbiota by treatment group and time point. Principal coordinate analysis (PCoA) plots based on Bray–Curtis (B) and Binary–Jaccard (C) distance matrices, illustrating the beta diversity of vaginal microbiota in the probiotic and placebo groups across the sampling times T1 and T2. Each point is a microbial community sample colored by its intervention group and sampling time. The PCoA plots show no significant (*p* > 0.05) clustering of samples according to treatment group or time point, suggesting that the overall microbial composition did not differ markedly between the treatment groups or sampling time. Figure S4: Upper panel: Relative abundance of bacterial phyla by individual sample across groups (placebo and probiotic) and sampling time points (T1 and T2) in vaginal exudate samples. Bar plots show the relative abundance of bacterial phyla, including *Firmicutes*, *Actinobacteriota*, *Proteobacteria* (now *Bacillota, Actinomycetota*, *Pseudomonadota*, respectively), *Bacteroidota*, *Fusobacteriota,* and Minor_phyla, across individual samples (left). Samples are organized by treatment group (probiotic [A] and placebo [B]) at both sampling times (T1 and T2). The hierarchical clustering heatmap (right) indicates the dominance of *Firmicutes* across most samples, with smaller contributions from other phyla. Lower panel: Relative abundance of major bacterial genera by individual sample across groups (placebo and probiotic) and sampling times (T1 and T2) in vaginal exudate samples. Bar plots illustrate the relative abundance of the most prominent bacterial genera, including *Lactobacillus*, *Gardnerella*, *Bifidobacterium*, *Atopobium*, *Anaerococcus*, and *Acinetobacter*, across individual samples (left). Samples are organized by treatment group (probiotic [A] and placebo [B]) at both sampling times (T1 and T2). The hierarchical clustering heatmap (right) highlights the consistent genus-level composition across samples. Figure S5: A: Boxplots showing the alpha diversity (Shannon and Simpson indices) of semen samples from the placebo (orange) and probiotic (blue) groups, at sampling time points 1 (light color) and 2 (dark color). Wilcoxon rank-sum tests were used to compare the change in alpha diversity indices before and after the intervention in the placebo and probiotic groups. B and C: Beta diversity (PCoA) of semen microbiota by treatment group and time point. Principal coordinate analysis (PCoA) plots based on Bray–Curtis (B) and Binary–Jaccard (C) distance matrices, illustrating the beta diversity of semen microbiota in the probiotic and placebo groups across the sampling times T1 and T2. The PCoA plots show no significant (*p* > 0.05) clustering of samples according to treatment group or time point, suggesting that the overall microbial composition did not differ markedly between the treatment groups or sampling time. Figure S6: Upper panel: Relative abundance of bacterial phyla by individual sample across groups (placebo and probiotic) and sampling time points (T1 and T2) in semen samples. Bar plots show the relative abundance of bacterial phyla, including *Firmicutes*, *Actinobacteriota* (now *Bacillota, Actinomycetota,* respectively), *Bacteroidota, Proteobacteria* (now *Pseudomonadota*), *Fusobacteriota*, and Minor_phyla, across individual samples. Samples are organized by probiotic (A) and placebo (B) groups at both sampling times (T1 and T2). The hierarchical clustering heatmap (right) indicates the dominance of *Firmicutes* across most samples, with variable contributions from other phyla. Lower panel: Relative abundance of major bacterial genera by individual sample across groups (placebo and probiotic) and sampling times (T1 and T2). Bar plots illustrate the relative abundance of the most prominent bacterial genera, including *Lactobacillus*, *Corynebacterium, Staphylococcus, Streptococcus,* and *Prevotella*, across individual samples. The data are categorized by probiotic (A) and placebo (B) groups at both sampling times (T1 and T2). The hierarchical clustering heatmap (right) highlights the variation in genus-level composition. Table S1: Baseline relevant clinical characteristics of female participants. Table S2: Baseline relevant clinical characteristics of male participants. Table S3: Pregnancy and birth information. Table S4: Taxonomic diversity of microbial isolates from vaginal exudate and semen samples. Table S5: Microbial diversity of vaginal exudate samples assessed using classical culture methods according to different outcomes of the study. Table S6: Prevalence and abundance of the main microbial genera in vaginal exudate samples assessed using classical culture methods according to the sampling time and intervention group. Table S7: Microbial diversity of semen samples assessed using classical culture methods according to different outcomes of the study. Table S8: Prevalence and abundance of the main microbial genera in semen samples assessed using classical culture methods according to the sampling time and intervention group.

## Abbreviations

The following abbreviations are used in this manuscript:

IVF	In vitro fertilization
TGFβ1	Transforming growth factor β1
VEGF	Vascular endothelial growth factor
IL	Interleukin
GM-CSF	Granulocyte macrophage colony-stimulating factor
IFNγ	Interferon γ
TNFα	Tumor necrosis factor α
