# Supplementary material for: Ligilactobacillus salivarius CECT5713 Increases Term Pregnancies in Women with Infertility of Unknown Origin: A Randomized, Triple-Blind, Placebo-Controlled Trial
Source: Nutrients. 2025 May 29;17(11):1860. doi: 10.3390/nu17111860 (PMC12158033; doi:10.3390/nu17111860)
Supplement: Supplementary file 1 [file nutrients-17-01860-s001.zip › Figure S1.pdf]

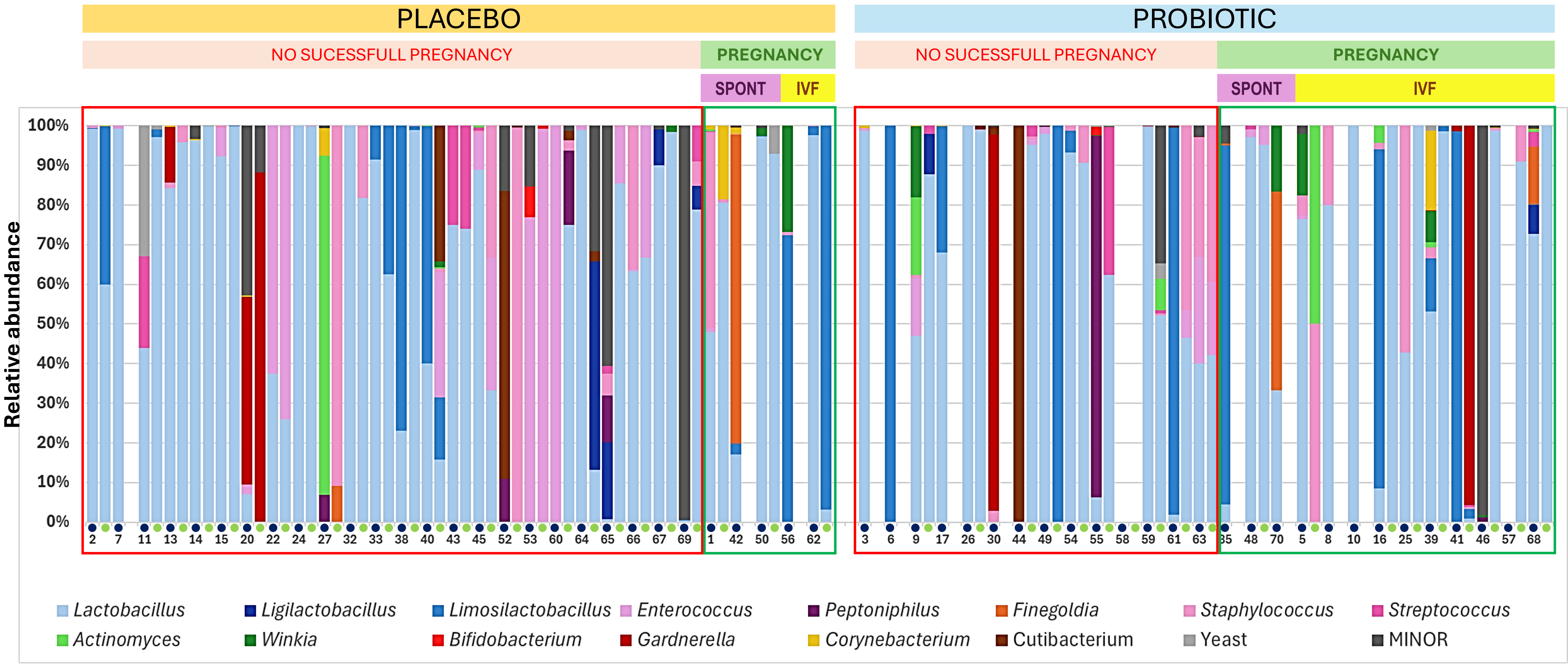
PLACEBO
PROBIOTIC
NO SUCESSFULL PREGNANCY
PREGNANCY
SPONT
IVF
Relative abundance
100%
90%
80%
70%
60%
50%
40%
30%
20%
10%
0%
2 7 11 13 14 15 20 22 24 27 32 33 38 40 43 45 52 53 60 64 65 66 67 69
1 42 50 56 62
3 6 9 17 26 30 44 49 54 55 58 59 61 63
35 48 70 5 8 10 16 25 39 41 46 57 68
Lactobacillus
Ligilactobacillus
Limosilactobacillus
Enterococcus
Peptoniphilus
Finegoldia
Staphylococcus
Streptococcus
Actinomyces
Winkia
Bifidobacterium
Gardnerella
Corynebacterium
Cutibacterium
Yeast
MINOR
