# Supplementary material for: Ligilactobacillus salivarius CECT5713 Increases Term Pregnancies in Women with Infertility of Unknown Origin: A Randomized, Triple-Blind, Placebo-Controlled Trial
Source: Nutrients. 2025 May 29;17(11):1860. doi: 10.3390/nu17111860 (PMC12158033; doi:10.3390/nu17111860)
Supplement: Supplementary file 1 [file nutrients-17-01860-s001.zip › Supplementary Tables.pdf]

Table S1. Baseline relevant clinical characteristics of female participants

	PLACEBO GROUP (N=30) n (%)	PROBIOTIC GROUP (N=27) n (%)	*p* value [1]
**Information about healthy habits**			
Alcohol consumption			
No	19 (63.3)	14 (51.9)	0.380*
Yes	11 (36.7)	13 (48.1)	
Occasional (<2 units/week)	11 (100.0)	10 (76.9)	0.223
Regularly (≥2 units/week)	-	3 (23.1)	
Smoker			
No	28 (93.3)	21 (77.8)	0.132
Yes	2 (6.7)	6 (22.2)	
Non-daily smoker (<1 cigarette/day)	1 (50.0)	1 (16.7)	1.000
Daily smoker (>1 cigarette/day)	1 (50.0)	5 (83.3)	
**Information about menstrual cycle**			
Age at menarche (years)	13.0 (12.0, 13.0)	13.0 (11.0, 14.0)	0.825
Regular menstrual cycle			
No	2 (6.7)	5 (18.5)	0.238
Yes	28 (93.3)	22 (81.5)	
Discomfort at any point in the menstrual cycle			
No	7 (23.3)	7 (25.9)	0.823*
Yes	23 (76.7)	20 (74.1)	
Yes, dysmenorrhea	17 (73.9)	18 (90.0)	0.228
Yes, during ovulation	1 (4.3)	1 (5.0)	
Yes, during the luteal phase	5 (21.7)	1 (5.0)	
**Information about reproductive health**			
Regular menstrual cycle			
No	2 (6.7)	5 (18.5)	0.238
Yes	28 (93.3)	22 (81.5)	
Children with another partner			
No	30 (100.0)	26 (96.3)	0.474
Yes	-	1 (3.7)	
**Medical history of infectious and autoimmune diseases and related treatments**			
Urinary tract infection			
No	8 (26.7)	23 (85.2)	0.697
Yes	22 (73.3)	4 (14.8)	
Recurrent	5 (22.7)	2 (11.8)	0.438
No recurrent	17 (77.3)	15 (88.2)	
Vaginal infection			
No	10 (33.3)	9 (33.3)	1.000*
Yes (mostly in adulthood)	20 (66.7)	18 (66.7)	
Recurrent	3 (15.0)	3 (16.7)	1.000
No recurrent	17 (85.0)	15 (83.3)	

Classified as General Business

Table S1 (cont.). Baseline relevant clinical characteristics of female participants

	PLACEBO GROUP (N=30) n (%)	PROBIOTIC GROUP (N=27) n (%)	*p* value[a]
**Medical history of infectious and autoimmune diseases and related treatments (cont.)**			
Causal agent of vaginal infection			
Unknown	2 (10.0)	3 (15.8)	0.841*
Known	18 (90.0)	16 (84.2)	
Candidiasis	18 (90.0)	16 (84.2)	-
Bacterial vaginosis	-	-	
The vaginal infection was related to			
Antibiotic treatment	5	7	
Stress	5	4	
Sexual intercourse	2	2	
Other	2	2	
Unknown	6	4	
Sexual transmitted infection			
No	25 (83.3)	22 (81.5)	1.000
Yes (*Chlamydia*, *Candida*, HPV)	5 (16.7)	5 (18.5)	
Treatment for genitourinary infections			
No	6 (20.0)	5 (18.5)	0.888*
Yes	24 (80.0)	22 (81.5)	
Treatment for genitourinary infections			
Antibiotic	18 (75.0)	13 (59.1)	0.729
Antifungal	17 (70.8)	18 (81.8)	
Antiviral	-	1 (4.5)	
Probiotic	1 (4.2)	1 (4.5)	
Oral infections			
No	20 (66.7)	22 (81.5)	0.204
Yes	10 (33.3)	5 (18.5)	
Respiratory tract infections			
No	23 (76.7)	22 (81.5)	0.655*
Yes	7 (23.2)	5 (18.5)	
GIT infections			
No	26 (86.7)	19 (70.4)	0.132*
Yes	4 (13.3)	8 (29.6)	
Skin infections			
No	28 (93.3)	25 (92.6)	1.000
Yes	2 (6.7)	2 (7.4)	
Autoimmune diseases			
No	25 (83.3)	25 (92.6)	0.427
Yes	5 (16.7)	2 (7.4)	
Allergies/intolerances			
No	14 (46.7)	14 (51.9)	0.698*
Yes	16 (53.3)	13 (48.1)	
Endocrine disorders			
No	25 (83.3)	25 (92.6)	
Yes	5 (16.7)	2 (7.4)	0.427

Classified as General Business

Table S1 (cont.). Baseline relevant clinical characteristics of female participants

	PLACEBO GROUP (N=30) n (%)	PROBIOTIC GROUP (N=27) n (%)	*p* value[a]
**Medical history of infectious and autoimmune diseases and related treatments (cont.)**			
Antibiotic use			
No (never)	3 (10.0)	1 (3.7)	0.613
Yes [2]	27 (90.0)	26 (96.3)	
Corticosteroids use			
No (never)	19 (63.3)	18 (66.7)	0.791*
Yes [2]	11 (36.7)	9 (33.3)	
Other diseases			
No	25	1920	0.393*
Yes	5	7	
Surgery during infancy			
No	25 (83.3)	21 (77.8)	0.597*
Yes	5 (16.7)	6 (22.2)	
Surgery during adulthood			
No	14 (46.7)	11 (40.7)	0.654*
Yes	16 (53.3)	16 (59.3)	

GIT, gastrointestinal; HPV, human papilloma virus; N, total number of participants in the group; n, number of participants with the specific characteristic described.

[1] Differences between groups (placebo, probiotic) were tested using the Chi-square contingency test (marked with an asterisk) or Fisher's Exact Probability test.

[2] A low frequency of use was noted in most instances.

Classified as General Business

Table S2. Baseline relevant clinical characteristics of male participants

	PLACEBO GROUP (N=30) n (%)	PROBIOTIC GROUP (N=27) n (%)	*p*-value [1]
**Information about healthy habits**			
Alcohol consumption			
No	14 (46.7)	5 (18.5)	0.024*
Yes	16 (53.3)	22 (81.5)	
Occasional (<2 units/week)	13 (81.3)	16 (72.7)	0.706
Regularly (≥2 units/week)	3 (18.7)	6 (27.3)	
Smoker			
No	26 (86.7)	20 (74.1)	0.228*
Yes	4 (13.3)	7 (25.9)	
Nondaily smoker (<1 cigarette/day)	2 (50.0)	1 (14.3)	0.491
Daily smoker (>1 cigarette/day)	2 (50.0)	6 (85.7)	
**Information about reproductive health**			
Previous kids with another partner			
No	27 (90)	25 (92.6)	1.000
Yes	3 (10)	2 (7.4)	
Abortions with another partner			
No	26 (86.7)	27 (100)	0.114
Yes	4 (13.3)	-	
**Medical history of infectious and autoimmune diseases and related treatments**			
Urinary tract infection			
No	27 (90)	23 (85.2)	0.697
Yes	3 (10)	4 (14.8)	
Prostate problems			
No	30 (100)	27 (100)	-
Yes	0	0	
Sexual transmitted infection			
No	30 (100)		0.100
Yes (*Chlamydia*, *Candida*, HPV)	0		
Oral infections			
No	22 (73.3)	23 (85.2)	0.273*
Yes	8 (26.7)	4 (14.8)	
Respiratory tract infections			
No	25 (83.3)	23 (85.2)	1.000
Yes	5 (16.7)	4 (14.8)	
GIT infections			
No	28 (93.3)	24 (88.9)	0.659
Yes	2 (6.7)	3 (11.1)	
Skin infections			
No	26 (86.7)	25 (92.6)	0.673
Yes	4 (13.3)	2 (7.4)	
Total infectious diseases			
No	18 (60.0)	13 (48.1)	0.371*
Yes	12 (40.0)	14 (51.9)	

Classified as General Business

Table S2 (cont.). Baseline relevant clinical characteristics of male participants

	PLACEBO GROUP (N=30) n (%)	PROBIOTIC GROUP (N=27) n (%)	*p*-value [1]
**Medical history of infectious and autoimmune diseases and related treatments (cont.)**			
Autoimmune diseases			
No	28 (93.3)	26 (96.3)	1.000
Yes	2 (6.7)	1 (3.7)	
Allergies/intolerances			
No	16 (53.3)	19 (70.4)	0.187*
Yes	14 (46.7)	8 (29.6)	
Antibiotic use			
No (never)	2 (6.7)	2 (7.4)	1.000
Yes [2]	28 (93.3)	25 (92.6)	
Corticosteroids use			
No (never)	23 (76.7)	19 (70.4)	0.590*
Yes [2]	7 (23.3)	8 (7.4)	
Other diseases			
No	23 (76.7)	24 (88.9)	0.304
Yes	7 (23.3)	3 (11.1)	

GIT, gastrointestinal; HPV, human papilloma virus; N, total number of participants in the group; n, number of participants with the specific characteristic described.

[1] Differences between groups (placebo, probiotic) were tested using the Chi-square contingency test (marked with an asterisk) or Fisher's Exact Probability test.

[2] A low frequency of use was noted in most instances.

Classified as General Business

Table S3. Pregnancy and birth information

	PLACEBO GROUP (N=6) n (%) or median (Q1, Q3) [1]	PROBIOTIC GROUP (N=13) n (%) or median (Q1, Q3)	*p*-value [2]
Complications during pregnancy			
No	3 (66.7)	5 (38.5)	1.000
Yes [3]	3 (33.3)	8 (53.8)	
GBS test			
Negative	5 (83.3)	13 (100.0)	0.316
Positive	1 (16.7)	0	
Delivery [4]			
Vaginal	5 (83.3)	8 (53.8)	0.605
C-Section	1 (16.7)	5 (38.5)	
Sex			
Girl	1 (16.7)	5 (30.8)	0.605
Boy	5 (83.3)	8 (61.5)	
Apgar score			
<8/8	1	1	0.779
≥8/8	4	10	
Unknown	1	2	
Infant weight (g)	3240.0 (2945.0, 3600.0)	3255.0 (3100.0, 3520.0)	0.963
Infant length (cm)	51.0 (50.0, 52.0)	50.0 [5] (48.0, 51.0)	0.162
Neonate problems			
No	5 (83.3)	9 (61.5)	0.631
Yes [7]	1 (16.7)	4 (30.8)	

GBS, Group B Streptococci

[1] All variables are expressed as the number (%) of individuals except infant weight and age that are expressed as medians (Q1, Q3).

[2] Differences between groups (placebo, probiotic) were tested using Fisher's Exact Probability test for categorical data and the Wilcoxon Rank-Sum test for continuous data.

[3] The reported complications during pregnancy in the placebo group included gestational anemia, hypothyroidism, vitamin D deficiency, and diabetes in one participant. Another woman in the placebo group exhibited increased nuchal translucency during gestation, which was subsequently determined to be non-pathological. Additionally, a third woman in the placebo group experienced gestational anemia. In the probiotic group, complications comprised gestational diabetes (n=3), gestational anemia (n=1) pathological uterine arteries (n=1), cervical shortening (n=1), intrahepatic cholestasis of pregnancy and large for gestational age fetus (n =1) and preeclampsia (=1).

[4] In the placebo group, two vaginal deliveries were expedited with a vacuum extractor (ventouse). In the probiotic group, one vaginal delivery underwent labor induction. In two cases of the probiotic group the expulsion stage was abbreviated, with forceps used in one instance. The sole C-section in the placebo group was conducted due to placenta accreta. In the probiotic group, three indications were identified: cephalo-pelvic disproportion (n=2), abnormal presentation (n=1) and seizure (n=1), and in one case, the specific reason for performing the C-section was not documented.

[5] Infant length information is missing for one case.

[6] Problems in newborns included neonatal sepsis caused by *Proteus vulgaris* (n=1), transient hyperbilirubinemia, which resolved soon afterwards (n=1), hemolytic hyperbilirubinemia due to isoinmmunization (n=1), and respiratory distress requiring admission (n=1) in the probiotic group, and macrodactyly of the index finger of the right hand (n=1) in the placebo group.

Table S4. Taxonomic diversity of microbial isolates from vaginal exudate and semen samples.

Phylum	Family [1]	Genus	Vaginal exudate n (%) [2]	Semen n (%)
Actinomycetota	*Bifidobacteriaceae*	*Alloscardovia*		2 (0.4)
	Bifidobacteriaceae	*Bifidobacterium*	6 (1.4)	
	Bifidobacteriaceae	*Gardnerella*	6 (1.4)	
	Micrococcaceae	*Rothia*		13 (2.7)
	Micrococcaceae	***Kocuria***	1 (0.2)	6 (1.2)
	Micrococcaceae	***Micrococcus***	2 (0.5)	10 (2.1)
	Micrococcaceae	*Arthrobacter*		1 (0.2)
	Micrococcaceae	*Pseudarthrobacter*	3 (0.7)	
	Dermabacteraceae	*Dermabacter*		4 (0.8)
	Dermabacteraceae	*Dermacoccus*		3 (0.6)
	Microbacteriaceae	*Gulosibacter*	1 (0.2)	
	Microbacteriaceae	***Microbacterium***	2 (0.5)	4 (0.8)
	Corynebacteriaceae	***Corynebacterium***	22 (5.0)	62 (12.9)
	Brevibacteriaceae	*Brevibacterium*	3 (0.7)	
	Propionibacteriaceae	*Cutibacterium*	20 (4.5)	
	Nocardiaceae	*Rhodococcus*		1 (0.2)
	Actinomycetaceae	***Actinomyces***	17 (3.8)	35 (7.3)
	Actinomycetaceae	*Gleimia*	1 (0.2)	
	Actinomycetaceae	***Schaalia***	6 (1.4)	3 (0.6)
	Actinomycetaceae	***Winkia***	10 (2.3)	31 (6.4)
	Actinomycetaceae	***Actinobaculum***	1 (0.2)	1 (0.2)
	Actinomycetaceae	***Actinotignum***	1 (0.2)	14 (2.9)
Total *Actinomycetota*			102 (23.1)	190 (39.5)
Bacillota	*Bacillaceae*	*Bacillus*		2 (0.4)
	Bacillaceae	*Exiguobacterium*	1 (0.2)	
	Staphylococcaceae	***Staphylococcus***	83 (18.8)	152 (31.6)
	Aerococcaceae	***Aerococcus***	4 (0.9)	22 (4.6)
	Aerococcaceae	***Facklamia***	5 (1.1)	6 (1.2)
	Aerococcaceae	*Abiotrophia*		1 (0.2)
	Enterococcaceae	***Enterococcus***	38 (8.6)	36 (7.5)
	Lactobacillaceae	***Lactobacillus*** [3]	155 (35.1)	17 (3.5)
	Lactobacillaceae	*Pediococcus*		1 (0.2)
	Streptococcaceae	***Streptococcus***	17 (3.8)	49 (10.2)
	Clostridiales Family XVII	*Thermaerobacter*		1 (0.2)
	Peptoniphilaceae	*Anaerococcus*	4 (0.9)	
	Peptoniphilaceae	*Finegoldia*	5 (1.1)	
	Peptoniphilaceae	*Peptoniphilus*	9 (2.0)	
	Peptostreptococcaceae	*Peptostreptococcus*	3 (0.7)	
	Selenomonadaceae	*Selenomonas*	1 (0.2)	
	Gemellaceae	*Gemella*		2 (0.4)
Total *Bacillota*			325 (73.5)	289 (60.1)
Pseudomonadota	*Enterobacteriaceae*	*Enterobacter*	1 (0.2)	
	Enterobacteriaceae	*Escherichia*	3 (0.7)	
	Moraxellaceae	*Acinetobacter*	1 (0.2)	
Total *Pseudomonadota*			5 (1.1)	
Bacteroidota	*Porphyromonadaceae*	*Porphyromonas*	1 (0.2)	
**Yeast**			9 (2.0)	2 (0.4)

[1] Bacterial families and genera present only in vaginal exudate samples are in black, those present only in semen samples are in blue, and those present in both sample types are in bold black.

[2] Percentages may not total 100 due to rounding.

[3] *Lactobacillus* includes both current and former members of the *Lactobacillus* genus.

Classified as General Business

Table S5. Microbial diversity of vaginal exudate samples assessed using classical culture methods according to different outcomes of the study.

	n	Number of bacterial species per sample (n) Median (Q1, Q3)	*p*-value [1]	Bacterial counts (log_{10} CFU/mL) Mean (95% CI)	*p*-value	Shannon index Mean (95% CI)	*p*-value	Simpson index Median (Q1, Q3)	*p*-value
**ALL SAMPLES**	98	4 (2, 6)		5.88 (5.63 – 6.13)		0.56 (0.47 – 0.65)		1.48 (1.08, 2.00)	
**Time**									
Time 1	54	5 (3, 8)	0.030	6.17 (5.86 – 6.49)	0.009	0.60 (0.48 – 0.72)	0.278	1.61 (1.08, 2.00)	0.409
Time 2	44	3 (2, 5)		5.52 (5.13 – 5.91)		0.51 (0.38 – 0.34)		1.25 (1.08, 1.97)	
**TIME 1**	54								
**Group**									
Placebo	29	5 (3, 8)	0.916	6.49 (6.09 – 6.88)	0.032	0.59 (0.45 – 0.74)	0.863	1.61 (1.22, 1.93)	0.986
Probiotic	25	4 (3, 9)		5.81 (5.31 – 6.31)		0.61 (0.41 – 0.82)		1.47 (1.05, 2.08)	
**Embryo transfer**									
No	24	5 (3, 7)	0.745	5.96 (5.43 – 6.49)	0.227	0.66 (0.45 – 0.88)	0.368	1.59 (1.10, 2.36)	0.548
Yes	30	4.5 (2, 8)		6.35 (5.94 – 6.75)		0.55 (0.41 – 0.70)		1.64 (1.05, 1.95)	
Fresh	7	5 (4, 8)	0.335	6.92 (6.20 – 7.65)	0.107	0.67 (0.48 – 0.87)	0.356	1.61 (1.35, 1.95)	0.695
Frozen	23	3 (2, 8)		6.17 (5.69 – 6.65)		0.52 (0.34 – 0.70)		1.67 (1.03, 1.96)	
**Pregnancy**									
No	34	5 (3, 8)	0.731	6.32 (5.89 – 6.76)	0.229	0.58 (0.41 – 0.75)	0.606	1.58 (1.03, 1.95)	0.441
Yes	20	4.5 (3, 8)		5.92 (5.46 – 6.39)		0.64 (0.48 – 0.81)		1.70 (1.28, 2.06)	
Spontaneous	6	5 (3, 8)	0.933	5.20 (4.10 – 6.29)	0.027	0.68 (0.37 – 0.99)	0.776	1.81 (1.21, 2.15)	0.650
After IVF	14	4.5 (2, 8)		6.24 (5.77 – 6.71)		0.63 (0.41 – 0.85)		1.70 (1.35, 1.96)	
**Successful pregnancy**									
No	37	5 (3, 8)	0.993	6.35 (5.95 – 6.74)	0.111	0.59 (0.44 – 0.75)	0.808	1.60 (1.05, 1.95)	0.689
Yes	17	4 (3, 8)		5.80 (5.28 – 6.32)		0.62 (0.44 – 0.81)		1.67 (1.21, 2.03)	

Classified as General Business

Table S5 (cont). Microbial diversity of vaginal exudate samples assessed using classical culture methods according to different outcomes of the study.

		Number of bacterial species per sample (n) Median (Q1, Q3)	*p*-value	Bacterial counts (log_{10} CFU/mL) Mean (95% CI)	*p*-value	Shannon index Mean (95% CI)	*p*-value	Simpson index Median (Q1, Q3)	*p*-value
**TIME 2**	44								
**Group**									
Placebo	26	3 (2, 4)	0.428	5.39 (4.88 – 5.89)	0.419	0.51 (0.34 – 0.68)	0.964	1.35 (1.07, 1.94)	0.905
Probiotic	18	4 (2, 5)		5.71 (5.05 – 6.37)		0.50 (0.29 – 0.72)		1.22 (1.09, 2.00)	
**Embryo transfer**									
No	21	4 (3, 4)	0.381	5.60 (5.02 – 6.17)	0.702	0.69 (0.48 – 0.89)	0.005	1.62 (1.20, 2.46)	0.009
Yes	23	3 (2, 5)		5.45 (4.87 – 6.02)		0.34 (0.20 – 0.48)		1.10 (1.03, 1.67)	
Fresh	7	3 (2, 4)	0.493	5.84 (5.13 – 6.56)	0.351	0.40 (0.09 – 0.71)	0.558	1.20 (1.03, 1.94)	0.482
Frozen	16	3.5 (2, 5.5)		5.27 (4.47 – 6.07)		0.32 (0.14 – 0.49)		1.10 (1.02, 1.47)	
**Pregnancy**									
No	29	4 (3, 5))	0.157	5.54 (5.09 – 5.99)	0.623	0.56 (0.39 – 0.74)	0.045	1.62 (1.10, 2.35)	0.069
Yes	15	2 (2, 5)		5.33 (4.49 – 6.18)		0.31 (0.18 – 0.44)		1.15 (1.07, 1.46)	
Spontaneous	3	3 (2, 8)	0.411	6.03 (4.67 – 7.40)	0.397	0.39 (0.18 – 0.59)	0.541	1.20 (1.15, 1.46)	0.469
After IVF	12	2 (2, 4.5)		5.16 (4.48 – 5.84)		0.29 (0.19 – 0.40)		1.11 (1.05, 1.41)	
**Successful pregnancy**									
No	32	3.5 (2, 4.5)	0.619	5.49 (5.05 – 5.93)	0.845	0.58 (0.41 – 0.74)	0.071	1.56 (1.07. 2.32)	0.166
Yes	12	2.5 (2, 6)		5.58 (4.63 – 6.53)		0.32 (0.18 – 0.46)		1.18 (1.08, 1.35)	

CFU, colony forming units; IVF, In Vitro Fertilization.

[1] For each variable, differences between groups were tested using the Wilcoxon Rank-Sum test for non-normally distributed variables and one-way ANOVA when data followed a normal distribution.

Classified as General Business

Table S6. Prevalence and abundance of the main microbial genera in vaginal exudate samples assessed using classical culture methods according to the sampling time and intervention group.

	TIME 1			TIME 2		
	**PLACEBO GROUP (N=29)**	**PROBIOTIC GROUP (N=25)**	***p*-value [1]**	**PLACEBO GROUP (N=26)**	**PROBIOTIC GROUP (N=18)**	***p*-value**
Actinomycetota						
Bifidobacterium						
Prevalence, n (%)	1 (3.4)	3 (12)	-	1 (3.8)	0 (-)	-
Abundance [b]	6.70	3.18 (2.70, 6.02)	-	4.54		-
Gardnerella						
Prevalence, n (%)	2 (6.9)	2 (8.0)	0.326	1 (3.8)	1 (5.6)	-
Abundance	6.20 (5.70, 6.70)	5.29 (4.18, 6.40)	-	5.18	7.30	-
Actinomyces						
Prevalence, n (%)	6 (20.7)	6 (24.0)	0.764*	3 (11.5)	2 (11.1)	1.0
Abundance	3.39 (2.70, 4.00)	4.20 (3.40, 5.00)	0.375	2.70 (2.70, 3.00)	3.85 (3.00, 4.70)	0.224
Winkia						
Prevalence, n (%)	2 (6.9)	6 (24.0)	0.125	2 (7.7)	1 (5.6)	-
Abundance	5.44 (4.70, 6.18)	4.68 (4.00, 5.00)	0.617	3.2 (2.70, 3.70)	3.40	-
Corynebacterium						
Prevalence, n (%)	8 (27.6)	6 (24.0)	0.764*	2 (7.7)	1 (5.6)	-
Abundance	3.92 (3.20, 4.21)	3.65 (2.70, 4.88)	0.948	4.44 (2.70, 6.18)	4.00	-
Cutibacterium						
Prevalence, n (%)	4 (13.8)	6 (24.0)	0.485	4 (15.4)	3 (16.7)	1.0
Abundance	2.94 (2.70, 5.59)	3.0 (2.70, 3.70)	0.910	2.85 (2.70, 4.02)	3.48 (2.70, 4.70)	0.854
Bacillota						
Staphylococcus						
Prevalence, n (%)	14 (48.3)	13 (52.0)	0.791*	12 (46.2)	12 (66.7)	0.179*
Abundance	4.41 (3.66 – 5.17)	4.29 (3.75 – 4.83)	0.785	3.62 (3.06 – 4.17)	4.15 (3.36 – 4.93)	0.250
Former *Lactobacillus*						
Prevalence, n (%)	25 (86.2)	23 (92.0)	0.674*	22 (84.6)	17 (94.4)	0.389*
Abundance	6.15 (5.82 – 6.47)	5.59 (5.25 – 5.93)	0.101	5.16 (4.80 – 5.53)	5.68 (5.27 – 6.10)	0.188

Classified as General Business

Table S6 (cont.). Prevalence and abundance of the main microbial genera in vaginal exudate samples assessed using classical culture methods according to the sampling time and intervention group

	TIME 1			TIME 2		
	**PLACEBO GROUP (N=29)**	**PROBIOTIC GROUP (N=25)**	***p*-value [1]**	**PLACEBO GROUP (N=26)**	**PROBIOTIC GROUP (N=18)**	***p*-value**
Enterococcus						
Prevalence, n (%)	15 (51.7)	7 (28.0)	0.077*	9 (34.6)	7 (38.9)	0.777*
Abundance	5.05 (4.32 – 5.79)	4.09 (2.58 – 5.59)	0.154	3.96 (2.88 – 5.04)	4.07 (2.85 – 5.29)	0.957
Streptococcus						
Prevalence, n (%)	6 (20.7)	2 (8.0)	0.262	3 (11.5)	6 (33.3)	0.128
Abundance	3.66 (3.00, 5.30)	4.20 (2.70, 5.70)	0.867	4.18 (2.70, 6.54)	4.35 (3.30, 5.48)	0.897
Finegoldia						
Prevalence, n (%)	1 (3.4)	3 (12.0)	-	0 (-)	0 (-)	-
Abundance	5.40	3.18 (2.70 – 6.30)	-			
Peptoniphilus						
Prevalence, n (%)	3 (10.3)	4 (16.0)	0.692	1 (3.8)	0 (-)	-
Abundance	7.18 (6.00, 7.18)	4.35 (4.00, 6.02)	0.373	3.88	-	
**Yeast**						
Prevalence, n (%)	1 (3.4)	2 (8.0)	-	3 (11.5)	3 (16.7)	0.676
Abundance	4.18	4.35 (3.70, 5.00)	-	3.40 (3.00, 6.00)	2.70 (2.70, 4.40)	1.000
**Minor**						
Prevalence, n (%)	12 (41.4)	7 (28.0)	0.306*	3 (11.5)	4 (22.2)	0.419
Abundance	4.85 (3.33, 7.18)	3.85 (3.70, 5.00)	0.397	3.78 (2.70, 4.30)	3.94 (2.94, 5.02)	0.721

[1] Differences in the prevalence of the main bacterial genera between the placebo and probiotic groups were analyzed using Fisher's Exact Probability test or Chi-square contingency test (marked with one asterisk). For abundance, the Wilcoxon Rank-Sum test was used for non-normally distributed data, while one-way ANOVA was applied for normally distributed data.

[b] Abundance is expressed as mean (95% CI) or median (Q1, Q3) log_{10} CFU/mL.

Classified as General Business

Table S6 (cont.). Prevalence and abundance of the main microbial genera in vaginal exudate samples assessed using classical culture methods according to the sampling time and intervention group.

	**PLACEBO GROUP**			**PROBIOTIC GROUP**		
	**TIME 1 (N=29)**	**TIME 2 (N=26)**	***p*-value [1]**	**TIME 1 (N=25)**	**TIME 2 (N=18)**	***p*-value**
Actinomycetota						
Bifidobacterium						
Prevalence, n (%)	1 (3.4)	1 (3.8)	-	3 (12)	0 (-)	0.252
Abundance [b]	6.70	4.54	-	3.18 (2.70, 6.02)		-
Gardnerella						
Prevalence, n (%)	2 (6.9)	1 (3.8)	1.000	2 (8.0)	1 (5.6)	1.000
Abundance	6.20 (5.70, 6.70)	5.18	-	5.29 (4.18, 6.40)	7.30	-
Actinomyces						
Prevalence, n (%)	6 (20.7)	3 (11.5)	0.475	6 (24.0)	2 (11.1)	0.434
Abundance	3.39 (2.70, 4.00)	2.70 (2.70, 3.00)	0.225	4.20 (3.40, 5.00)	3.85 (3.00, 4.70)	0.737
Winkia						
Prevalence, n (%)	2 (6.9)	2 (7.7)	1.000	6 (24.0)	1 (5.6)	0.209
Abundance	5.44 (4.70, 6.18)	3.2 (2.70, 3.70)	0.245	4.68 (4.00, 5.00)	3.40	-
Corynebacterium						
Prevalence, n (%)	8 (27.6)	2 (7.7)	0.082	6 (24.0)	1 (5.6)	0.209
Abundance	3.92 (3.20, 4.21)	4.44 (2.70, 6.18)	0.895	3.65 (2.70, 4.88)	4.00	-
Cutibacterium						
Prevalence, n (%)	4 (13.8)	4 (15.4)	1.000	6 (24.0)	3 (16.7)	0.711
Abundance	2.94 (2.70, 5.59)	2.85 (2.70, 4.02)	0.878	3.0 (2.70, 3.70)	3.48 (2.70, 4.70)	0.685
Bacillota						
Staphylococcus						
Prevalence, n (%)	14 (48.3)	12 (46.2)	0.888*	13 (52.0)	12 (66.7)	0.337*
Abundance	4.41 (3.66 – 5.17)	3.62 (3.06 – 4.17)	0.086	4.29 (3.75 – 4.83)	4.15 (3.36 – 4.93)	0.739
Former *Lactobacillus*						
Prevalence, n (%)	25 (86.2)	22 (84.6)	1.000	23 (92.0)	17 (94.4)	1.000
Abundance	6.15 (5.82 – 6.47)	5.16 (4.80 – 5.53)	**0.005**	5.59 (5.25 – 5.93)	5.68 (5.27 – 6.10)	0.819

Classified as General Business

Table S6 (cont.). Prevalence and abundance of the main microbial genera in vaginal exudate samples assessed using classical culture methods according to the sampling time and intervention group.

	PLACEBO GROUP			PROBIOTIC GROUP		
	**TIME 1 (N=29)**	**TIME 2 (N=26)**	***p*-value** [1]	**TIME 1 (N=25)**	**TIME 2 (N=18)**	***p*-value**
Enterococcus						
Prevalence, n (%)	15 (51.7)	9 (34.6)	0.202	7 (28.0)	7 (38.9)	0.450*
Abundance	5.05 (4.32 – 5.79)	3.96 (2.88 – 5.04)	**0.070**	4.09 (2.58 – 5.59)	4.07 (2.85 – 5.29)	0.987
Streptococcus						
Prevalence, n (%)	6 (20.7)	3 (11.5)	0.475	2 (8.0)	6 (33.3)	**0.052**
Abundance	3.66 (3.00, 5.30)	4.18 (2.70, 6.54)	0.897	4.20 (2.70, 5.70)	4.35 (3.30, 5.48)	0.868
Finegoldia						
Prevalence, n (%)	1 (3.4)	0 (-)	-	3 (12.0)	0 (-)	0.252
Abundance	5.40		-	3.18 (2.70 – 6.30)		
Peptoniphilus						
Prevalence, n (%)	3 (10.3)	1 (3.8)	0.613	4 (16.0)	0 (-)	0.127
Abundance	7.18 (6.00, 7.18)	3.88	-	4.35 (4.00, 6.02)	-	
**Yeast**						
Prevalence, n (%)	1 (3.4)	3 (11.5)	0.334	2 (8.0)	3 (16.7)	0.634
Abundance	4.18	3.40 (3.00, 6.00)	-	4.35 (3.70, 5.00)	2.70 (2.70, 4.40)	0.773
**Minor**						
Prevalence, n (%)	12 (41.4)	3 (11.5)	**0.013***	7 (28.0)	4 (22.2)	1.000
Abundance	4.85 (3.33, 7.18)	3.78 (2.70, 4.30)	0.279	3.85 (3.70, 5.00)	3.94 (2.94, 5.02)	0.776

[1] Differences in the prevalence of the main bacterial genera between the placebo and probiotic groups were analyzed using Fisher's Exact Probability test or Chi-square contingency test (marked with one asterisk). For abundance, the Wilcoxon Rank-Sum test was used for non-normally distributed data, while one-way ANOVA was applied for normally distributed data.

[b] Abundance is expressed as mean (95% CI) or median (Q1, Q3) log_{10} CFU/mL.

Classified as General Business

Table S7. Microbial diversity of semen samples assessed using classical culture methods according to different outcomes of the study.

	n	Number of bacterial species per sample (n) Median (Q1, Q3)	*p*-value [1]	Bacterial counts (log_{10} CFU/mL) Mean (95% CI)	*p*-value	Shannon index Mean (95% CI)	*p*-value	Simpson index Median (Q1, Q3)	*p*-value
**ALL SAMPLES**	97	5 (4, 6)		4.15 (3.94 – 4.35)		0.95 (0.86 – 1.03)		2.18 (1.57, 2.88)	
**Time**									
Time 1	54	5 (4, 6)	0.494	4.26 (4.07 – 4.46)	0.221	0.96 (0.88 – 1.05)	0.641	2.21 (1.50, 2.93)	0.747*
Time 2	43	5 (4, 6)		4.01 (3.79 – 4.22)		0.92 (0.83 – 1.01)		2.14 (1.57, 2.87)	
**TIME 1**	54								
**Group**									
Placebo	28	5.5 (4, 6)	0.979	4.40 (4.14 – 4.66)	0.289	0.93 (0.81 – 1.05)	0.568	2.16 (1.47, 2.88)	0.616
Probiotic	26	5 (4, 6)		4.12 (3.85 – 4.39)		1.00 (0.87 – 1.13)		2.33 (1.79, 3.09)	
**Embryo transfer**									
No	24	5 (4, 6)	0.811	4.21 (3.93 – 4.50)	0.737	0.97 (0.84 – 1.10)	0.971	2.12 (1.49, 2.98)	0.993
Yes	30	4.5 (4, 7)		4.30 (4.05 – 4.56)		0.96 (0.84 – 1.08)		2.22 (1.63, 2.88)	
Fresh	8	5 (4, 7)	0.686	4.29 (3.81 – 4.78)	0.977	0.94 (0.70 – 1.18)	0.890	2.16 (1.96, 2.82)	0.870
Frozen	22	4 (3.5, 6.5)		4.31 (4.02 – 4.60)		0.97 (0.82 – 1.12)		2.32 (1.47, 2.88)	
**Pregnancy**									
No	33	4 (3, 6)	0.070	4.08 (3.84 – 4.31)	0.080	0.89 (0.78 – 1.00)	0.144	1.99 (1.47 – 2.93)	0.260*
Yes	21	6 (4, 7)		4.55 (4.26 – 4.85)		1.08 (0.94 – 1.21)		2.45 (2.12 – 2.88)	
Spontaneous	6	5.5 (5, 7)	0.905	4.20 (3.68 – 4.72)	0.250	1.33 (1.09 – 1.57)	0.078	3.15 (2.45, 3.78)	0.080
After IVF	15	6 (4, 7)		4.69 (4.36 – 5.02)		0.97 (0.82 – 1.13)		2.26 (1.28, 2.45)	
**Successful pregnancy**									
No	36	4.5 (3.5, 6)	0.215	4.19 (3.96 – 4.42)	0.445	0.89 (0.78 – 0.99)	0.075	2.06 (1.47 – 2.88)	0.123*
Yes	18	5.5 (4, 7)		4.41 (4.08 – 4.73)		1.12 (0.97 – 1.26)		2.73 (2.15 – 3.64)	

Classified as General Business

Table S7 (cont.). Microbial diversity of semen samples assessed using classical culture methods according to different outcomes of the study

		Number of bacterial species per sample (n) Median (Q1, Q3)	*p*-value[b]	Bacterial counts (log cfu/mL) Mean (95% CI)	*p*-value[b]	Shannon index Mean (95% CI)	*p*-value[b]	Simpson index Median (Q1, Q3)	*p*-value
**TIME 2**	43	4.8 (4.2 – 5.3)		4.01 (3.68 – 4.33)		0.92 (0.80 – 1.04)		2.27 (2.01 -2.53)	
**Group**									
Placebo	24	5 (4, 6)	0.403	4.01 (3.70 – 4.32)	0.972	1.01 (0.90 – 1.12)	0.099	2.34 (1.76, 2.89)	0.167
Probiotic	19	4 (3, 6)		4.00 (3.65 – 4.35)		0.81 (0.68 – 0.94)		2.05 (1.30, 2.71)	
**Embryo transfer**									
No	21	4 (4, 6)	0.386	3.88 (3.55 – 4.21)	0.457	0.90 (0.78 – 1.03)	0.739	2.30 (1.66, 2.85)	0.884
Yes	22	5 (4, 6)		4.13 (3.80 – 4.45)		0.94 (0.82 – 1.06)		2.09 (1.57, 2.90)	
Fresh	7	4 (3, 6)	0.174	3.82 (3.25 – 4.40)	0.362	0.74 (0.53 – 0.95)	0.104	2.09 (1.17, 2.37)	0.290
Frozen	15	5 (4, 6)		4.27 (3.87 – 4.66)		1.04 (0.89 – 1.18)		2.58 (1.61, 2.99)	
**Pregnancy**									
No	28	4.5 (4, 6)	0.363	3.76 (3.48 – 4.03)	**0.032**	0.90 (0.79 – 1.01)	0.611	2.22 (1.61, 2.86)	0.959
Yes	15	5 (4, 6)		4.48 (4.10 – 4.85)		0.97 (0.82 – 1.11)		2.09 (1.57, 2.90)	
Spontaneous	3	6 (5, 8)	0.181	4.94 (4.06 – 5.82)	0.384	1.10 (0.80 – 1.40)	0.453	1.85 (1.57, 4.17)	0.885
After IVF	12	4.5 (4, 6)		4.36 (3.92 – 4.80)		0.93 (0.78 – 1.08)		2.34 (1.59, 2.81)	
**Successful pregnancy**									
No	31	5 (4, 6)	0.956	3.90 (3.63 – 4.17)	0.277	0.91 (0.81 – 1.02)	0.802	2.14 (1.65, 2.87)	0.989
Yes	12	4.5 (4, 6)		4.29 (3.86 – 4.73)		0.95 (0.78 – 1.11)		2.22 (1.57, 2.85)	

CFU, colony forming units; IVF, In Vitro Fertilization.

[1] For each variable, differences between groups were tested using the Wilcoxon Rank-Sum test for non-normally distributed variables and one-way ANOVA when data followed a normal distribution.

Classified as General Business

Table S8. Prevalence and abundance of the main microbial genera in semen samples assessed using classical culture methods according to the sampling time and intervention group.

	TIME 1			TIME 2		
	**PLACEBO GROUP (N=28)**	**PROBIOTIC GROUP (N=26)**	***p*-value [a]**	**PLACEBO GROUP (N=24)**	**PROBIOTIC GROUP (N=19)**	***p*-value**
Actinomycetota						
Rothia						
Prevalence, n (%)	2 (37.1)	1 (3.9)	1.000	5 (20.8)	4 (21.1)	1.000
Abundance [b]	3.35 (3.00, 3.70)	4.18	-	2.70 (2.60, 4.00)	2.85 (2.20, 3.50)	0.902
Kocuria						
Prevalence, n (%)	0	2 (7.7)	0.227	2 (8.3)	2 (10.5)	1.000
Abundance	-	1.70 (1.7, 1,7)	-	2.44 (2.18, 2.70)	2.20 (1.70, 2,70)	0.712
Micrococcus						
Prevalence, n (%)	3 (10.7)	3 (11.5)	1.000	2 (8.3)	1 (5.3)	1.000
Abundance	2.70 (2.18, 4.00)	3.00 (2.00, 3.33)	0.787	1.85 (1.70, 2.00)	2.00	-
Corynebacterium						
Prevalence, n (%)	12 (42.9)	16 (61.5)	0.170*	13 (54.2)	9 (47.4)	0.654*
Abundance	3.36 (2.70, 4.30)	3.29 (2.70, 3.78)	0.292	3.19 (2.70, 3.90)	3.30 (2.65, 4.18)	0.947
Actinomyces						
Prevalence, n (%)	8 (28.6)	10 (38.5)	0.442*	8 (33.3)	4 (21.1)	0.374*
Abundance	4.09 (3.70, 5.00)	3.35 (3.00, 3.88)	0.109	3.55 (3.00, 3.85)	4.05 (2.55, 4.80)	0.865
Winkia						
Prevalence, n (%)	10 (35.7)	9 (34.6)	0. 920*	7 (29.2)	3 (15.8)	0.470
Abundance	3.85 (2.78, 4.10)	4.70 (3.70, 4.78)	0.286	4.00 (3.15, 5.00)	4.7 (3.00, 5.88)	0.546
Actinogtignum						
Prevalence, n (%)	4 (14.3)	3 (11.5)	1.000	2 (8.3)	5 (26.3)	0.211
Abundance	3.94 (3.33, 5.30)	3.65 (3.30, 5.18)	0.810	4.00 (3.70, 5.30)	3.48 (3.48, 5.40)	0.817

Classified as General Business

Table S8 (cont). Prevalence and abundance of the main microbial genera in semen samples assessed using classical culture methods according to the sampling time and group

	TIME 1			TIME 2		
	**PLACEBO GROUP (N=28)**	**PROBIOTIC GROUP (N=26)**	***p*-value [1]**	**PLACEBO GROUP (N=24)**	**PLACEBO GROUP (N=19)**	***p*-value**
Bacillota						
Staphylococcus						
Prevalence, n (%)	25 (89.3)	21 (80.8)	0.460	20 (83.3)	15 (78.9)	1.000
Abundance	3.37 (2.91 – 3.83)	3.10 (2.84 – 3.36)	0.380	3.16 (2.78 – 3.53)	3.11 (2.80 – 3.42)	0.250
Aerococcus						
Prevalence, n (%)	6 (21.4)	3 (11.5)	0.470	6 (25.0)	7 (36.8)	0.399*
Abundance	3.18 (3.00, 3.70)	2.70 (2.70, 4.00)	0.679	2.94 (2.18, 3.70)	4.18 (2.70, 4.30)	0.163
Facklamia						
Prevalence, n (%)	2 (7.1)	2 (7.7)	1.000	2 (8.3)	0	0.495
Abundance	3.64 (3.40, 3.88)	3.74 (3.00, 4.48)	1.000	2.70 (2.70, 2.70)	-	-
Enterococcus						
Prevalence, n (%)	9 (32.1)	10 (38.5)	0.624*	10 (41.7)	7 (36.8)	0.752*
Abundance	2.69 (2.27, 3.11)	3.02 (2.68 – 3.36)	0.539	2.92 (2.52 3.32)	2.93 (2.52 – 3.34)	0.987
Lactobacillus						
Prevalence, n (%)	4 (14.3)	6 (23.1)	0.494	1 (4.2)	4 (21.1)	0.153
Abundance	3.55 (3.05, 3.85)	3.89 (2.70, 4.48)	0.883	1.70	3.05 (2.55, 4.05)	0.957
Streptococcus						
Prevalence, n (%)	12 (42.9)	9 (34.6)	0.532*	9 (37.5)	6 (31.6)	0.689*
Abundance	3.64 (2.53, 4.19)	2.87 (2.36, 3.39)	0.164	3.30 (2.78, 3.90)	3.14 (2.51, 3.77)	0.718
**Minor**						
Prevalence, n (%)	8 (28.6)	7 (26.9)	0.887*	3 (12.5)	4 (21.1)	0.680
Abundance	3.50 (2.70, 4.24)	2.30 (1.70, 5.00)	0.506	3.48 (2.78, 4.00)	3.65 (2.85, 4.45)	0.730

[1] Differences in the prevalence of the main bacterial genera between the placebo and probiotic groups were analyzed using Fisher's Exact Probability test or Chi-square contingency test (marked with one asterisk). For abundance, the Wilcoxon Rank-Sum test was used for non-normally distributed data, while one-way ANOVA was applied for normally distributed data.

[b] Abundance is expressed as mean (95% CI) or median (Q1, Q3) log_{10} CFU/mL.

Classified as General Business

Table S8 (cont). Prevalence and abundance of the main microbial genera in semen samples assessed using classical culture methods according to the sampling time and group

	Cambio en el tiempo					
	PLACEBO GROUP			PROBIOTIC GROUP		
	TIME 1 (N=28)	TIME 2 (N=24)	*p*-value [a]	TIME 1 (N=26)	TIME 2 (N=19)	*p*-value
Actinomycetota						
Rothia						
Prevalence, n (%)	2 (37.1)	5 (20.8)	0.227	1 (3.9)	4 (21.1)	0.146
Abundance [b]	3.35 (3.00, 3.70)	2.70 (2.60, 4.00)	0.846	4.18	2.85 (2.20, 3.50)	-
Kocuria						
Prevalence, n (%)	0	2 (8.3)	0.208	2 (7.7)	2 (10.5)	1.000
Abundance	-	2.44 (2.18, 2.70)	-	1.70 (1.7, 1,7)	2.20 (1.70, 2,70)	0.617
Micrococcus						
Prevalence, n (%)	3 (10.7)	2 (8.3)	1.000	3 (11.5)	1 (5.3)	0.627
Abundance	2.70 (2.18, 4.00)	1.85 (1.70, 2.00)	0.149	3.00 (2.00, 3.33)	2.00	-
Corynebacterium						
Prevalence, n (%)	12 (42.9)	13 (54.2)	0.417*	16 (61.5)	9 (47.4)	0.345*
Abundance	3.36 (2.70, 4.30)	3.19 (2.70, 3.90)	0.827	3.29 (2.70, 3.78)	3.30 (2.65, 4.18)	0.671
Actinomyces						
Prevalence, n (%)	8 (28.6)	8 (33.3)	0.708*	10 (38.5)	4 (21.1)	0.213*
Abundance	4.09 (3.70, 5.00)	3.55 (3.00, 3.85)	0.153	3.35 (3.00, 3.88)	4.05 (2.55, 4.80)	0.478
Winkia						
Prevalence, n (%)	10 (35.7)	7 (29.2)	0.617	9 (34.6)	3 (15.8)	0.158*
Abundance	3.85 (2.78, 4.10)	4.00 (3.15, 5.00)	0.591	4.70 (3.70, 4.78)	4.7 (3.00, 5.88)	1.000
Actinogtignum						
Prevalence, n (%)	4 (14.3)	2 (8.3)	0.674	3 (11.5)	5 (26.3)	0.253
Abundance	3.94 (3.33, 5.30)	4.00 (3.70, 5.30)	0.817	3.65 (3.30, 5.18)	3.48 (3.48, 5.40)	0.880

Classified as General Business

Table S8 (cont). Prevalence and abundance of the main microbial genera in semen samples assessed using classical culture methods according to the sampling time and group

	**PLACEBO GROUP**			**PROBIOTIC GROUP**		
	**TIME 1 (N=28)**	**TIME 2 (N=24)**	***p*-value [1]**	**TIME 1 (N=26)**	**TIME 2 (N=19)**	***p*-value**
Bacillota						
Staphylococcus						
Prevalence, n (%)	25 (89.3)	20 (83.3)	0.690	21 (80.8)	15 (78.9)	1.000
Abundance	3.37 (2.91 – 3.83)	3.16 (2.78 – 3.53)	0.482	3.10 (2.84 – 3.36)	3.11 (2.80 – 3.42)	0.973
Aerococcus						
Prevalence, n (%)	6 (21.4)	6 (25.0)	0.764*	3 (11.5)	7 (36.8)	0.070
Abundance	3.18 (3.00, 3.70)	2.94 (2.18, 3.70)	0.628	2.70 (2.70, 4.00)	4.18 (2.70, 4.30)	0.354
Facklamia						
Prevalence, n (%)	2 (7.1)	2 (8.3)	1.000	2 (7.7)	0	0.501
Abundance	3.64 (3.40, 3.88)	2.70 (2.70, 2.70)	0.221	3.74 (3.00, 4.48)	-	-
Enterococcus						
Prevalence, n (%)	9 (32.1)	10 (41.7)	0.475	10 (38.5)	7 (36.8)	0.920*
Abundance	2.69 (2.27, 3.11)	2.92 (2.52 3.32)	0.555	3.02 (2.68 – 3.36)	2.93 (2.52 – 3.34)	0.800
Lactobacillus						
Prevalence, n (%)	4 (14.3)	1 (4.2)	0.358	6 (23.1)	4 (21.1)	1.000
Abundance	3.55 (3.05, 3.85)	1.70	-	3.89 (2.70, 4.48)	3.05 (2.55, 4.05)	0.831
Streptococcus						
Prevalence, n (%)	12 (42.9)	9 (37.5)	0.698*	9 (34.6)	6 (31.6)	0.823*
Abundance	3.64 (2.53, 4.19)	3.30 (2.78, 3.90)	0.670	2.87 (2.36, 3.39)	3.14 (2.51, 3.77)	0.628
**Minor**						
Prevalence, n (%)	8 (28.6)	3 (12.5)	0.157*	7 (26.9)	4 (21.1)	0.736
Abundance	3.50 (2.70, 4.24)	3.48 (2.78, 4.00)	0.919	2.30 (1.70, 5.00)	3.65 (2.85, 4.45)	0.342

[1] Differences in the prevalence of the main bacterial genera between the placebo and probiotic groups were analyzed using Fisher's Exact Probability test or Chi-square contingency test (marked with one asterisk). For abundance, the Wilcoxon Rank-Sum test was used for non-normally distributed data, while one-way ANOVA was applied for normally distributed data.

[b] Abundance is expressed as mean (95% CI) or median (Q1, Q3) log_{10} CFU/mL.

Classified as General Business
