# Supplementary figures and images for: Ligilactobacillus salivarius CECT5713 Increases Term Pregnancies in Women with Infertility of Unknown Origin: A Randomized, Triple-Blind, Placebo-Controlled Trial
Source: Nutrients. 2025 May 29;17(11):1860. doi: 10.3390/nu17111860 (PMC12158033; doi:10.3390/nu17111860)

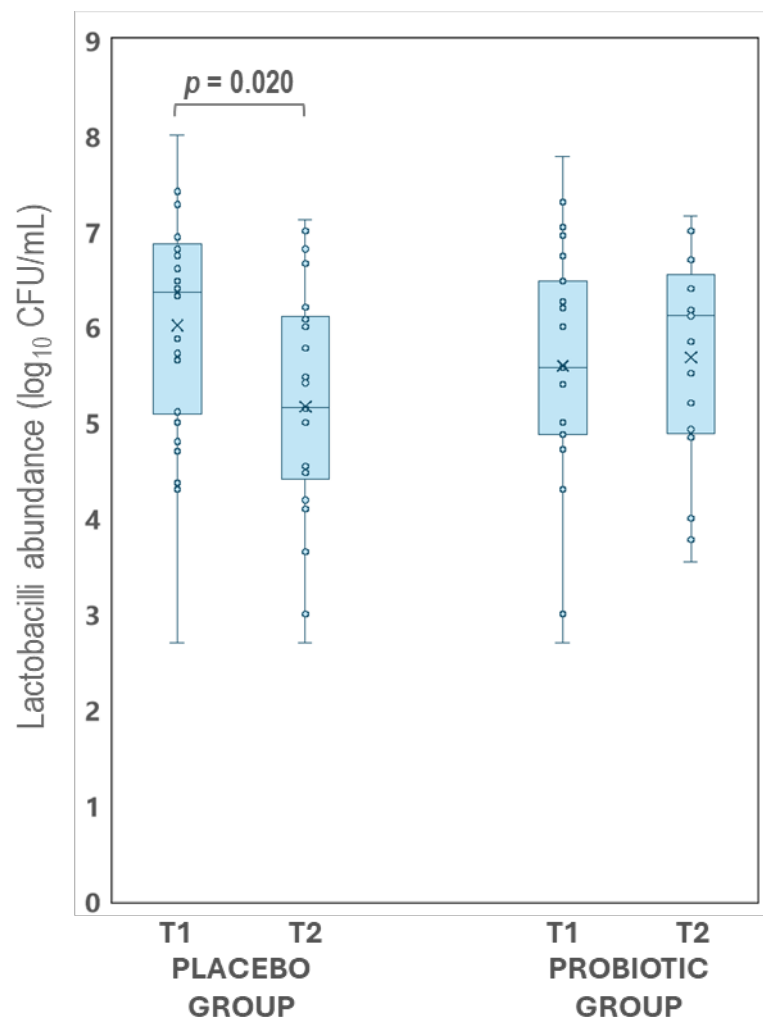

Supplement: Supplementary file 1 [file nutrients-17-01860-s001.zip › Figure S2.pdf]

**A**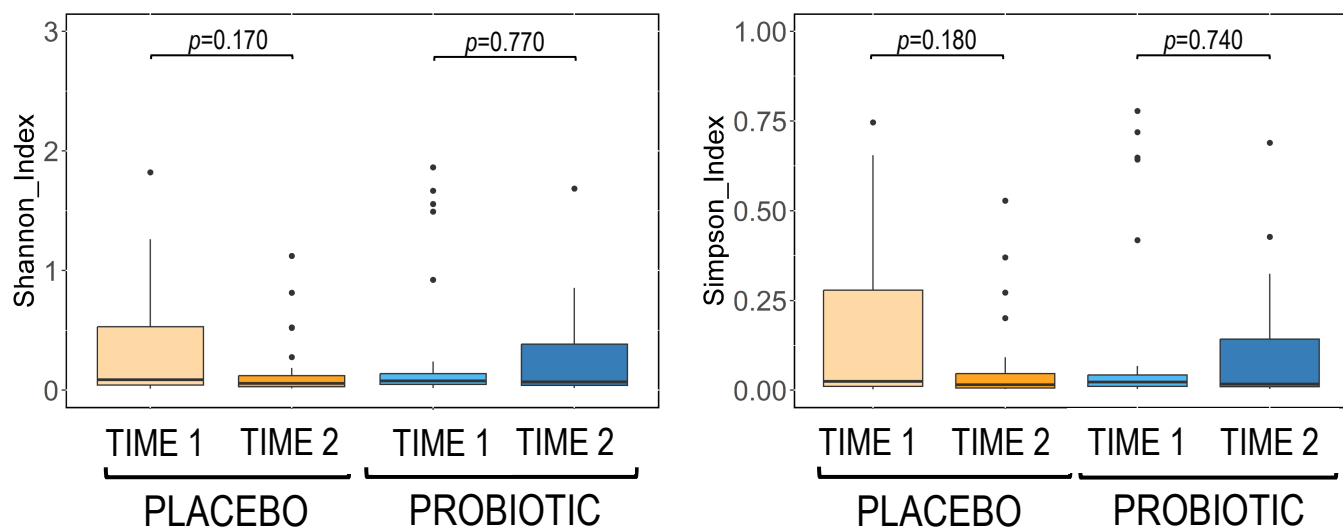**B****BRAY-CURTIS**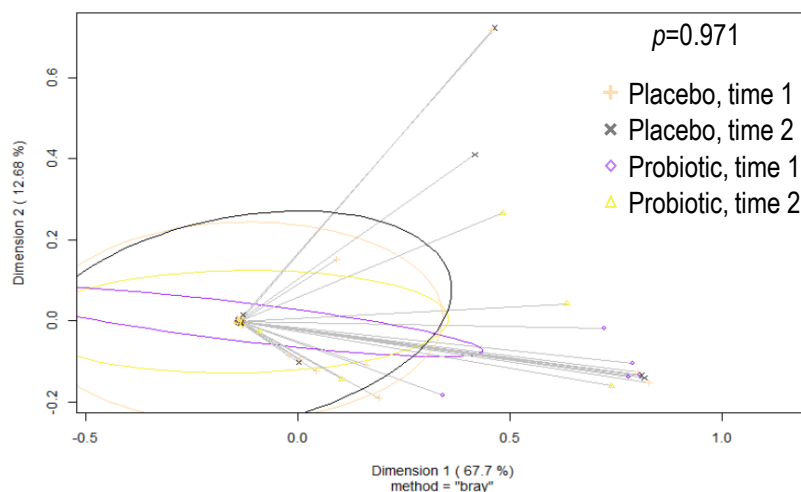**C****JACCARD**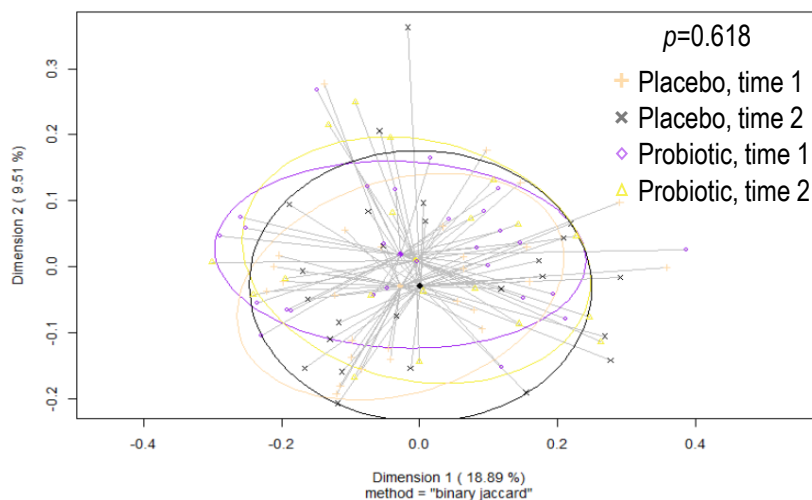

Supplement: Supplementary file 1 [file nutrients-17-01860-s001.zip › Figure S3.pdf]

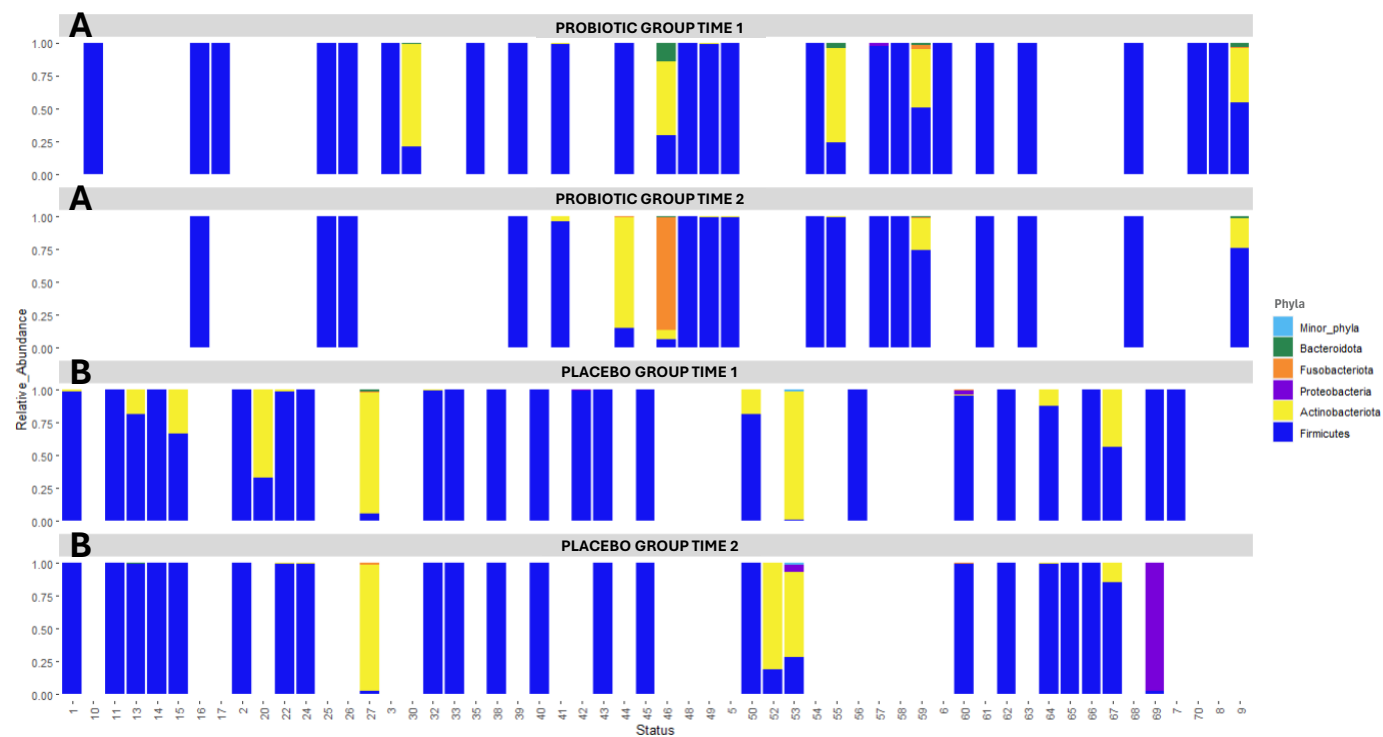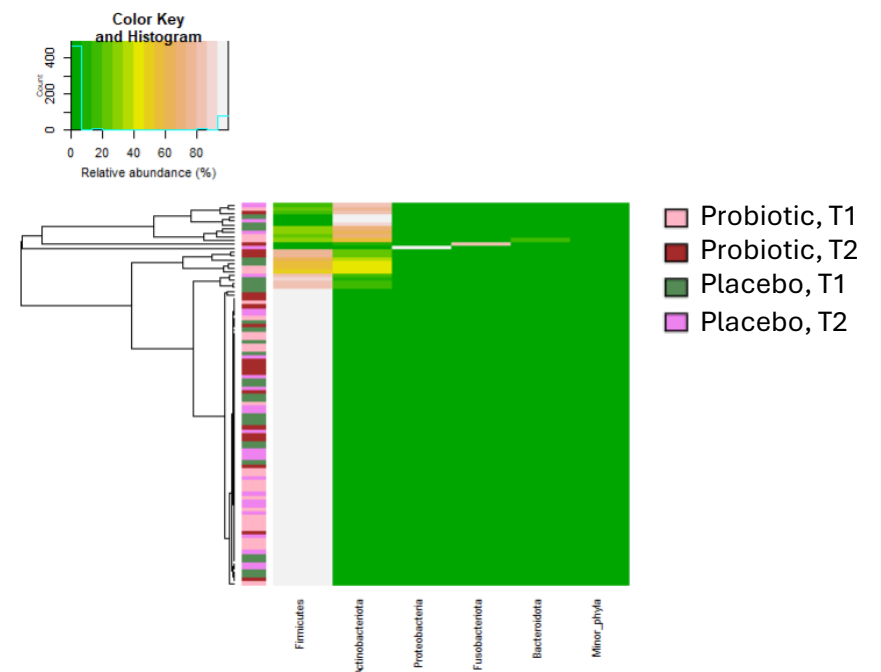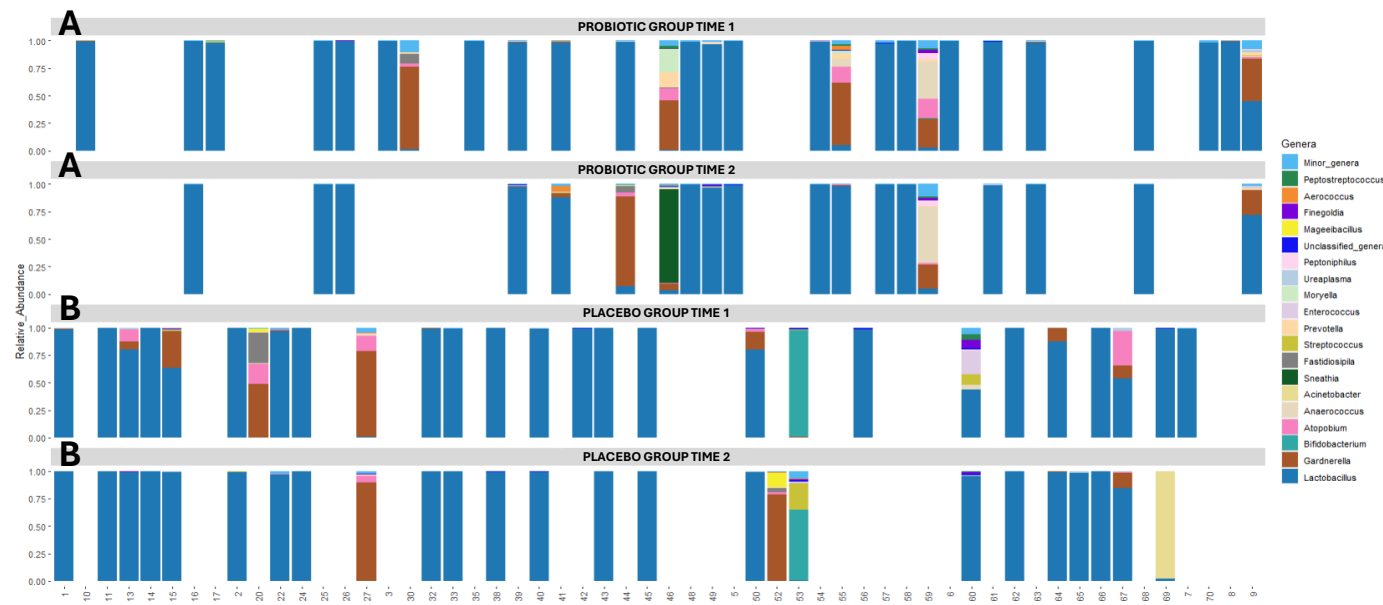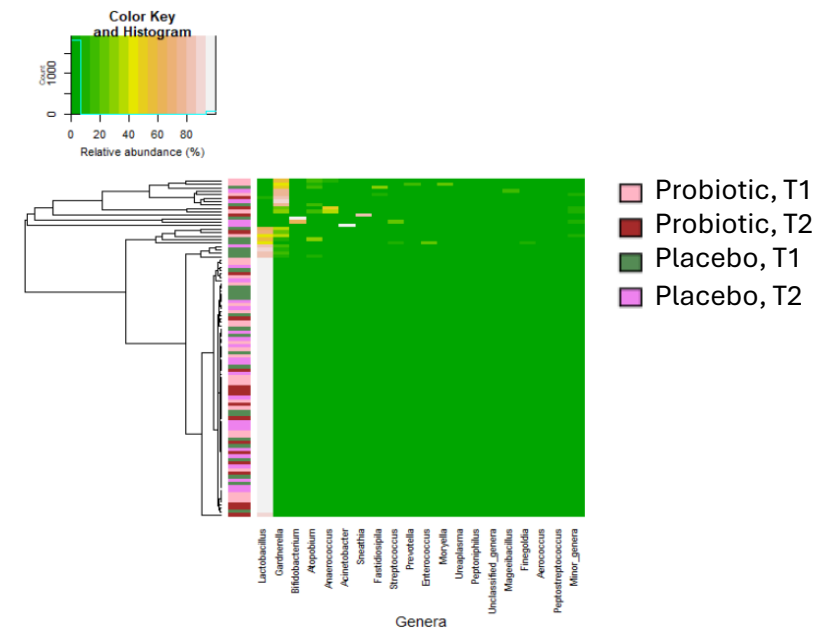

Supplement: Supplementary file 1 [file nutrients-17-01860-s001.zip › Figure S4.pdf]

**A**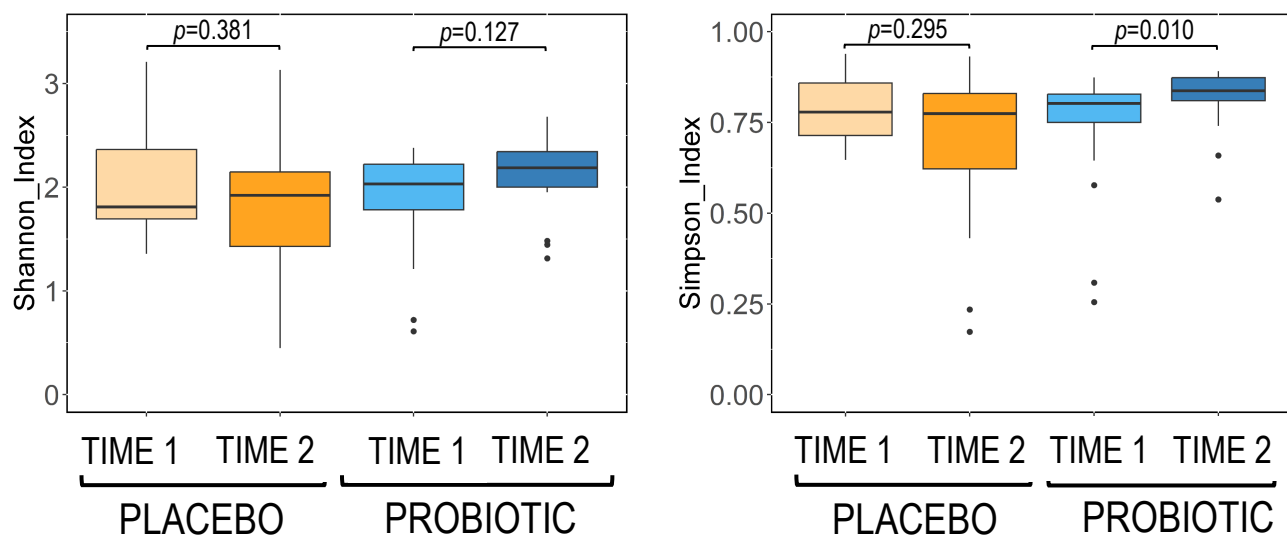**B****BRAY-CURTIS**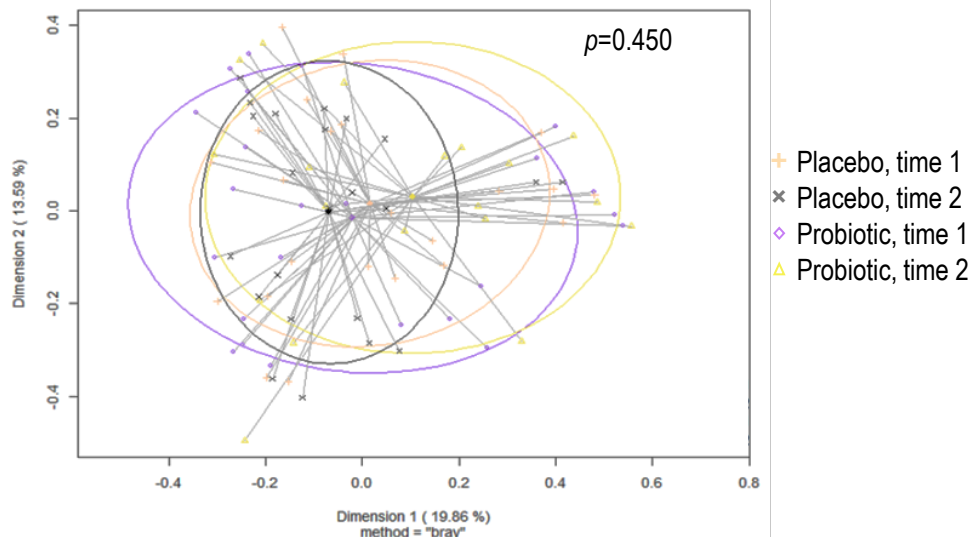**C****JACCARD**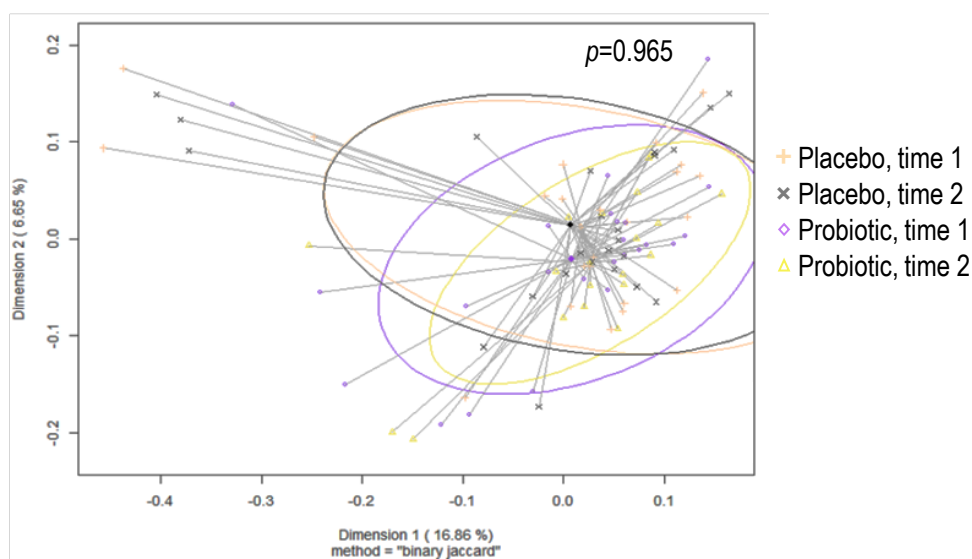

Supplement: Supplementary file 1 [file nutrients-17-01860-s001.zip › Figure S5.pdf]

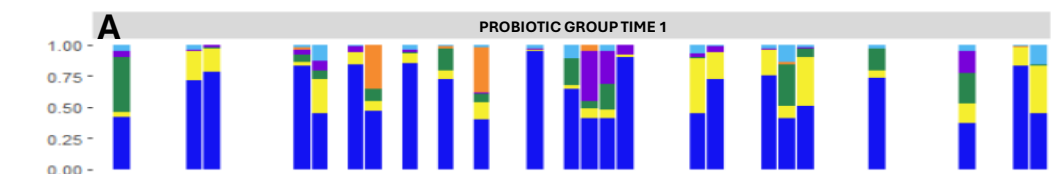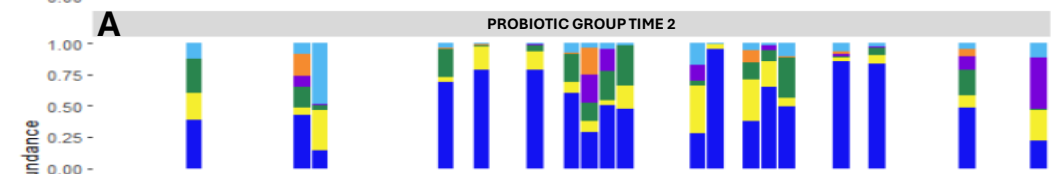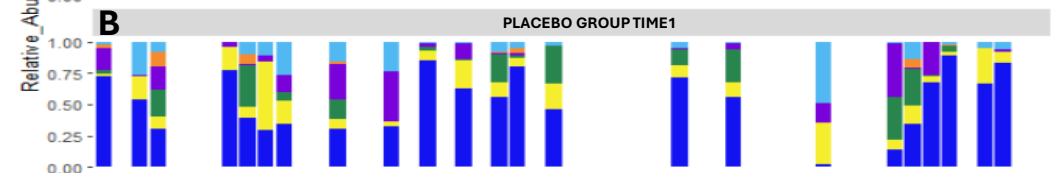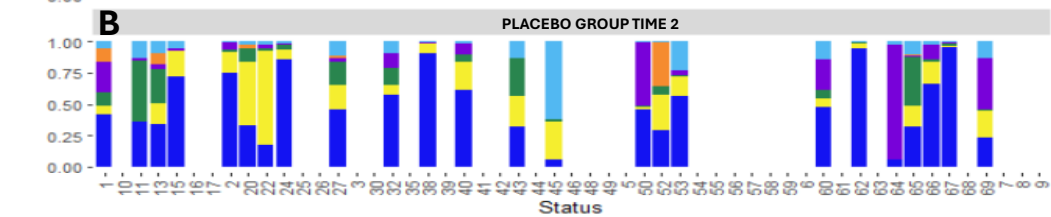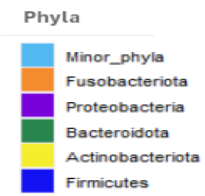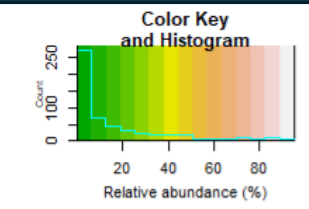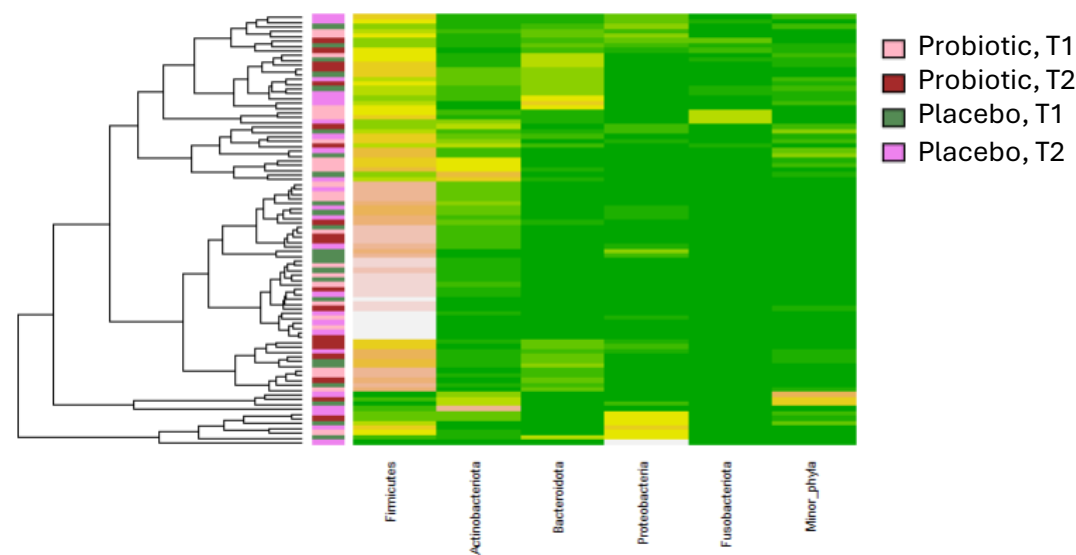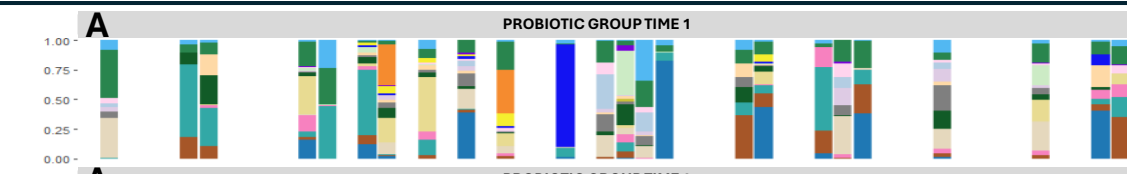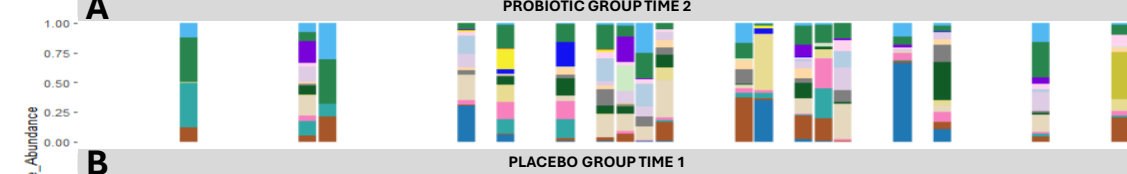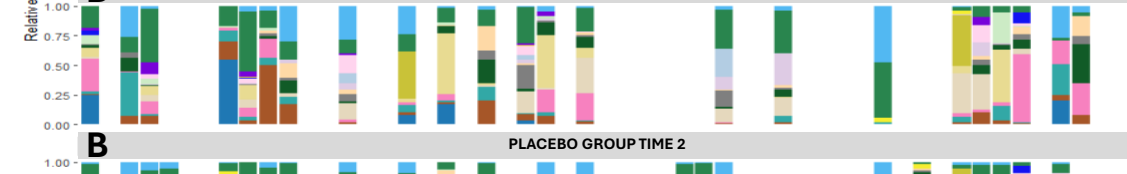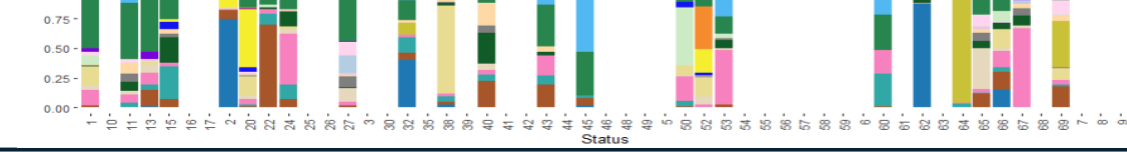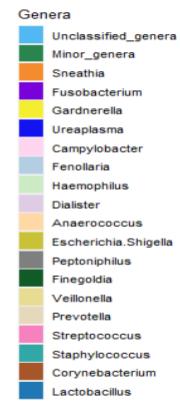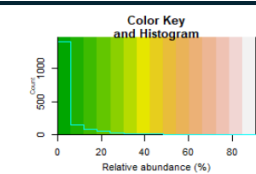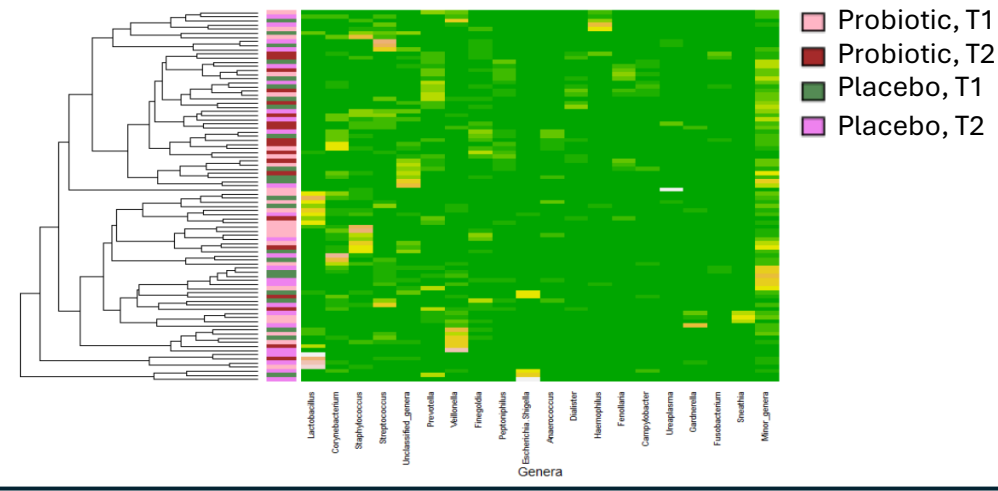

Supplement: Supplementary file 1 [file nutrients-17-01860-s001.zip › Figure S6.pdf]
